# Upregulation of miR-181a impairs hepatic glucose and lipid homeostasis

**DOI:** 10.18632/oncotarget.20523

**Published:** 2017-08-24

**Authors:** Xiliang Du, Yuchen Yang, Chuang Xu, Zhicheng Peng, Min Zhang, Lin Lei, Wenwen Gao, Yuhao Dong, Zhen Shi, Xudong Sun, Zhe Wang, Xiaobing Li, Xinwei Li, Guowen Liu

**Affiliations:** ^1^ Key Laboratory of Zoonosis, Ministry of Education, College of Veterinary Medicine, Jilin University, Changchun 130062, China; ^2^ College of Animal Science and Veterinary Medicine, Heilongjiang Bayi Agricultural University, Daqing 163319, China

**Keywords:** non-alcoholic fatty liver disease, dairy cows, non-esterified fatty acids, miR-181a, sirtuin1

## Abstract

The contributions of altered post-transcriptional gene silencing to the development of metabolic disorders remain poorly understood thus far. The objective of this study was to evaluate the roles of miR-181a in the regulation of hepatic glucose and lipid metabolism. MiR-181a is abundantly expressed in the liver, and we found that blood and hepatic miR-181a levels were significantly increased in patients and dairy cows with non-alcoholic fatty liver disease, as well as in high-fat diet and ob/ob mice. We determined that sirtuin1 is a target of miR-181a. Moreover, we found that hepatic sirtuin1 and peroxisome proliferator-activated receptor-γ coactivator-1α expression levels are downregulated, and acetylated peroxisome proliferator-activated receptor-γ coactivator-1α expression levels are upregulated in patients and dairy cows with non-alcoholic fatty liver disease, as well as in high-fat diet and ob/ob mice. MiR-181a overexpression inhibits the sirtuin1-peroxisome proliferator-activated receptor-γ coactivator-1α pathway, reduces insulin sensitivity, and increases gluconeogenesis and lipid synthesis in dairy cow hepatocytes and HepG2 cells. Conversely, silencing of miR-181a over-activates the sirtuin1-peroxisome proliferator-activated receptor-γ coactivator-1α pathway, increases insulin sensitivity and glycogen content, and decreases gluconeogenesis and lipid synthesis in hepatocytes, even under non-esterified fatty acids treatment conditions. Furthermore, miR-181a overexpression or sirtuin1 knockdown in mice increases lipid accumulation and decreases insulin sensitivity and glycogen content in the liver. Taken together, these findings indicate that increased hepatic miR-181a impairs glucose and lipid homeostasis by silencing sirtuin1 in non-alcoholic fatty liver disease.

## INTRODUCTION

Non-alcoholic fatty liver disease (NAFLD) is a major metabolic disorder of animals and humans and affects up to 30% of adults and 10% of children in developed countries [1]. The disease develops when hepatic lipid uptake exceeds hepatic lipid oxidation and secretion [2, 3]. NAFLD is the hepatic manifestation of the metabolic syndrome and is characterized by steatosis, insulin resistance, oxidative stress, inflammation and apoptosis [1, 4, 5]. In the first month after calving, 5 to 10% of dairy cows have severe NAFLD, and 30 to 40% have moderate fatty liver, indicating that up to 50% of dairy cows are at high risk for diseases and reproductive problems [6]. One reason is that dietary intake is insufficient to meet the increased requirements of energy for maintenance and lactation [7, 8]. More importantly, NAFLD has a great economic impact on herds, as the disease decreases milk yield, increases the incidence of infectious diseases and the risk of culling, and increases the time to conception [9, 10]. Previous studies showed that NAFLD is strongly associated with increased levels of serum non-esterified fatty acids (NEFA) [11, 12], which participate in many important cellular processes, such as energy storage, cellular membrane synthesis, and intracellular signaling pathways. However, chronic elevations in NEFA levels are capable of disturbing multiple metabolic pathways and inducing insulin resistance, endoplasmic reticular stress, oxidative stress, and mitochondrial dysfunction [13–15].

MicroRNAs (miRNAs), which were discovered by Ambros in 1993, are group of endogenous small noncoding RNAs ranging from 21-25 nucleotides in length [16]. Previous studies have identified several miRNAs whose expression is increased or decreased in human NAFLD or experimental NAFLD induced by dietary or genetic manipulations [17, 18]. Furthermore, a previous study showed that expression of miR-26a, an obesity-regulated miRNA, is regulated by NEFA in human adipocytes [19].

Mammalian sirtuins (SIRT1-7) are a group of NAD^+^-dependent protein deacetylases [20]. Accumulating evidence indicates that these enzymes are involved in a wide variety of cellular processes related to energy metabolism, tumorigenesis, stress responses and aging [21–23]. SIRT1 is the most conserved sirtuin protein and has emerged as a key metabolic sensor that regulates glucose and lipid metabolism in the liver, fat mobilization in white adipose tissue and insulin secretion in the pancreas [24]. Prolonged fasting leads to increases in SIRT1 activity and activation of peroxisome proliferator-activated receptor-γ coactivator-1α (PGC-1α) and the lipid-sensing nuclear receptor peroxisome proliferator-activated receptor (PPARα), resulting in increased fatty acid oxidation and improved glucose homeostasis [25].

Examination of the miRBase database (http://microrna.sanger.ac.uk/) showed that four miR-181s, namely, miR-181a, miR-181b, miR-181c, and miR-181d, have been identified previously. Studies have shown that miR-181a is highly expressed in many tissues, such as the thymus, brain and liver [26, 27], and that miR-181a suppresses SIRT1 protein expression in HEK293T and mouse embryonic stem cells [28, 29]. However, the biological function of miR-181a in NAFLD has not been reported in the literature. In the present work, we identified SIRT1 as a target of miR-181a and provide both *in vivo* and *in vitro* data demonstrating that hepatic miR-181a is a core regulator of hepatic glucose and lipid metabolism. In addition, we aim to provide clinicians with potential approaches for combating metabolic dysfunction in dairy cows and humans with NAFLD.

## RESULTS

### MiR-181a expression is increased in patients, mice and cows with NAFLD

**Figure 1:**
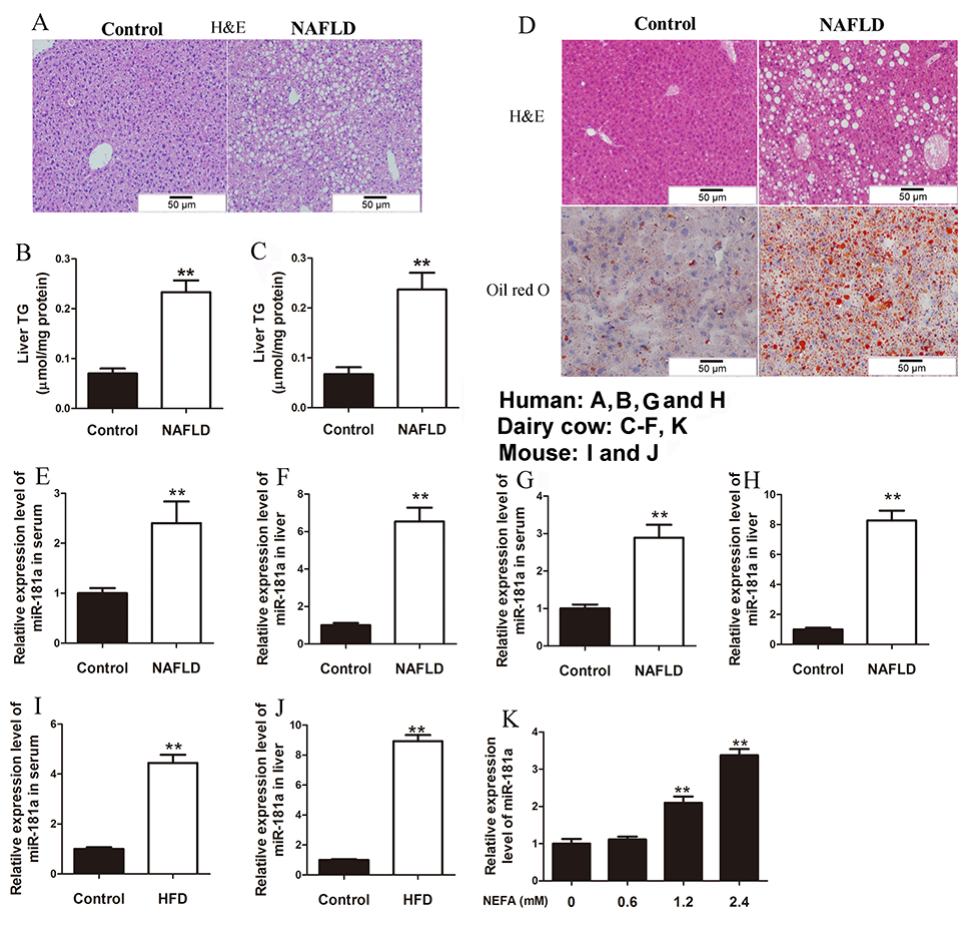
MiR-181a expression is increased in patients, mice and cows with NAFLD **(A)** Representative images of H&E staining (original magnification × 20) of liver sections from patients with NAFLD and controls. **(B)** The TG content in the liver of patients with NAFLD (*n* = 25) and controls (*n* = 15). **(C)** The TG content in the liver of dairy cows with NAFLD (*n* = 20) and controls (*n* = 20). **(D)** Representative images of H&E and Oil-red O staining (original magnification × 20) of liver sections from dairy cows with NAFLD and controls. **(E)** The expression level of miR-181a in the serum of dairy cows with NAFLD (*n* = 20) and controls (*n* = 20). **(F)** The expression level of miR-181a in the liver of dairy cows with NAFLD (*n* = 20) and controls (*n* = 20). **(G)** The expression level of miR-181a in the serum of patients with NAFLD (*n* = 25) and controls (*n* = 15). **(H)** The expression level of miR-181a in the liver of patients with NAFLD (*n* = 25) and controls (*n* = 15). **(I)** The expression level of miR-181a in the serum of HFD (*n* = 7) and control (*n* = 7) mice. **(J)** The expression level of miR-181a in the liver of HFD (*n* = 7) and control (*n* = 7) mice. **(K)** Relative expression level of miR-181a in hepatocytes. The hepatocytes were treated with 0, 0.6, 1.2 or 2.4 *mM* NEFA. ^*^P < 0.05, ^**^P < 0.01. All experiments were repeated at least three times and representative results are shown.

As shown in Supplementary Table 1, patients with NAFLD displayed high blood NEFA, glucose, insulin, HbA_1c_, triglyceride (TG), alanine aminotransferase (ALT), aspartate transaminase (AST) and gamma-glutamyl transpeptidase (γ-GT) levels. In addition, the body mass index (BMI) and homeostasis model assessment of insulin resistance (HOMA-IR) were significantly higher in patients with NAFLD than in control subjects. These data demonstrate that patients with NAFLD display significant systemic insulin resistance and hyperlipidemia. Besides, our hepatic H&E staining results, as well as our observation of elevated hepatic TG content in patients with NAFLD, indicate that these patients display significant hepatic lipid accumulation compared with control subjects (Figure 1A and 1B).

As shown in Supplementary Table 2, the body weights and body condition scores of NAFLD cows were significantly higher than those of control cows. In addition, blood NEFA, glucose, insulin, ALT, AST and γ-GT levels were significantly increased in NAFLD cows compared with control cows (Supplementary Table 2). Western blotting results showed that insulin receptor (IR), protein kinase B (Akt), and glycogen synthase kinase-3β (GSK3β) phosphorylation levels and PPARα protein expression levels were significantly decreased, and sterol regulator element-binding protein-1c (SREBP-1c) protein expression levels were significantly increased in the liver samples from dairy cows with NAFLD compared with those from control cows (Supplementary Figure 1A and 1B). SREBP-1c is a master regulator of the lipogenic pathway [30], and PPARα is a key transcription factor that regulates lipid oxidation gene expression [31]. Moreover, elevated hepatic TG content, H&E and Oil Red O staining demonstrated that the livers of dairy cows with NAFLD exhibited significant lipid accumulation compared with those of control cows (Figure 1C and 1D). These data suggest that insulin sensitivity is impaired and that lipid is accumulated in the livers of dairy cows with NAFLD.

We next measured miR-181a expression in the sera and livers of patients and dairy cows with NAFLD. The results showed that serum and hepatic miR-181a expression levels were significantly higher in patients and dairy cows with NAFLD, as well as in high-fat diet (HFD) and ob/ob mice (Figure 1E–1J; Supplementary Figure 2A and 2B). Excessive accumulation of NEFA in hepatocytes is a main pathological factor underlying the development of NAFLD in patients and dairy cows [2, 23]. Our results showed that high concentrations of NEFA (NEFA at concentrations ≥ 1.2 *mM*) impaired insulin signaling and increase lipid accumulation in the hepatocytes of dairy cows (Supplementary Figure 3A–3C). We further analyzed miR-181a expression in hepatocytes treated with different concentrations of NEFA. The results showed that NEFA dose-dependently elevated miR-181a expression in treated hepatocytes compared with control hepatocytes (Figure 1K).

Based on these data, we propose that miR-181a may be involved in regulating hepatic glucose and lipid metabolism.

### MiR-181a directly targets SIRT1

**Figure 2:**
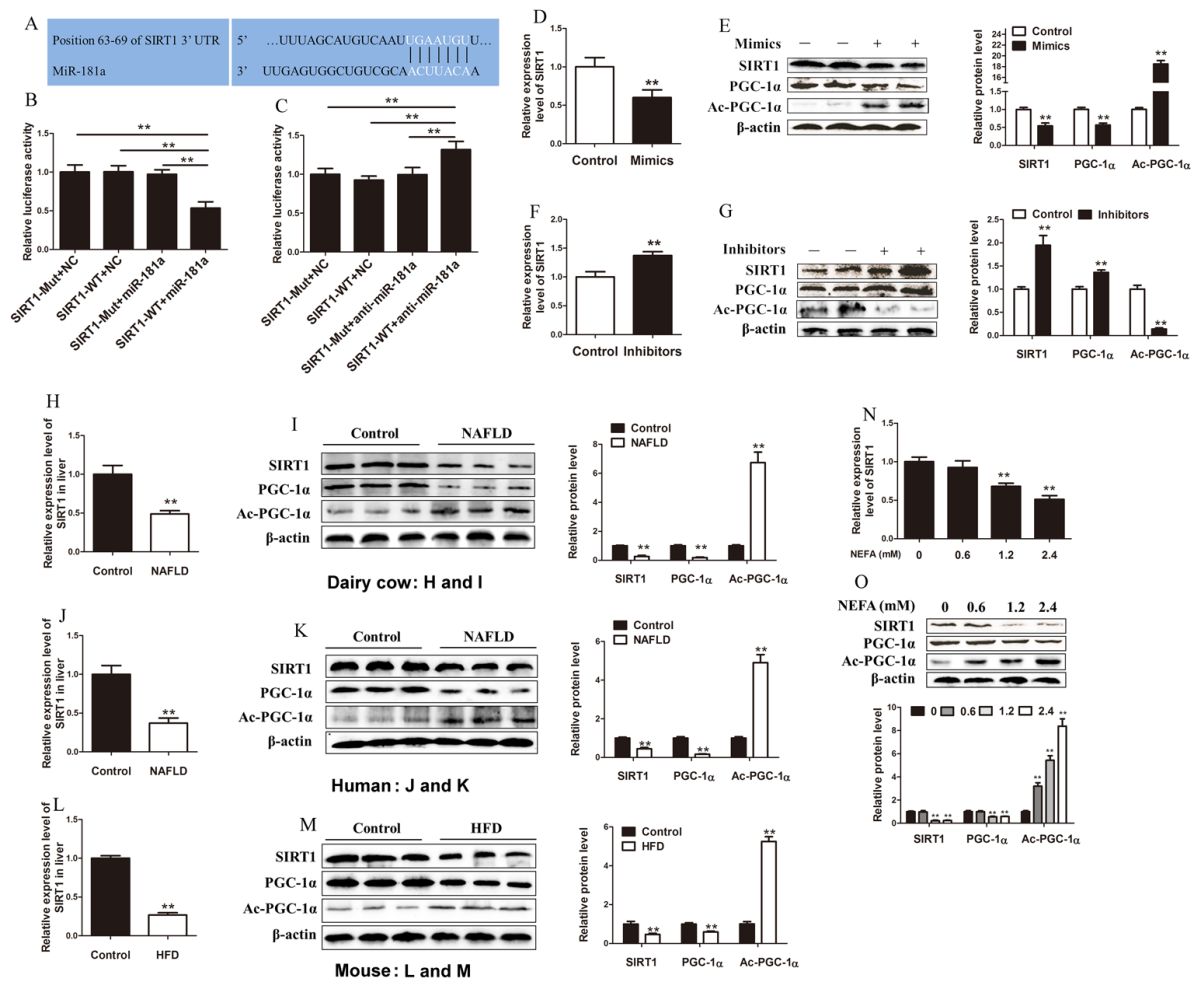
SIRT1 is a directly target of miR-181a **(A)** Schematic representation of the SIRT1 3’-UTR and the putative miR-181a target site. **(B)** Relative luciferase activity. Wild-type SIRT1 3’ -UTR reporter plasmids or mutated SIRT1 3’-UTR reporter plasmids were co-transfected with miR-181a mimic or the negative control. Firefly luciferase activity was standardized to the Renilla luciferase control. **(C)** Relative luciferase activity. Wild-type SIRT1 3’ -UTR reporter plasmids or mutated SIRT1 3’-UTR reporter plasmids were co-transfected with miR-181a inhibitor or the negative control. Firefly luciferase activity was standardized to the Renilla luciferase control. (D and E) Hepatocytes were transfected with 10 *nM* miR-181a mimics or negative control. **(D)** The mRNA expression level of SIRT1 in hepatocytes. **(E)** Immunoblot analysis (left) and quantification (right) of SIRT1, PGC-1α and acetylated PGC-1α in hepatocytes. (F and G) Hepatocytes were transfected with 50 *nM* miR-181a inhibitors or negative control. **(F)** The mRNA expression level of SIRT1 in hepatocytes. **(G)** Immunoblot analysis (left) and quantification (right) of SIRT1, PGC-1α and acetylated PGC-1α in hepatocytes. **(H)** The mRNA expression level of SIRT1 in the liver of dairy cows with NAFLD (*n* = 20) and controls (*n* = 20). **(I)** Immunoblot analysis (left) and quantification (right) of SIRT1, PGC-1α and acetylated PGC-1α in the liver of dairy cows with NAFLD (*n* = 20) and controls (*n* = 20). **(J)** The mRNA expression level of SIRT1 in the liver of patients with NAFLD (*n* = 25) and controls (*n* = 15). **(K)** Immunoblot analysis (left) and quantification (right) of SIRT1, PGC-1α and acetylated PGC-1α in the liver of patients with NAFLD (*n* = 25) and controls (*n* = 15). **(L)** The mRNA expression level of SIRT1 in the liver of HFD (*n* = 7) and control (*n* = 7) mice. **(M)** Immunoblot analysis (left) and quantification (right) of SIRT1, PGC-1α and acetylated PGC-1α in the liver of HFD (*n* = 7) and control (*n* = 7) mice. (N, O) The hepatocytes were treated with 0, 0.6, 1.2 or 2.4 *mM* NEFA. **(N)** Relative mRNA expression level of SIRT1 in hepatocytes. **(O)** Immunoblot analysis (top) and quantification (bottom) of SIRT1, PGC-1α and acetylated PGC-1α in hepatocytes. ^*^P < 0.05, ^**^P < 0.01. All experiments were repeated at least three times and representative results are shown.

Using TargetScan, we found that SIRT1 contains a potential miRNA response element (MRE) for miR-181a in its 3′-untranslated region (UTR; Figure 2A). To determine whether SIRT1 is a direct target of miR-181a, the 3’-UTR of the SIRT1 mRNA, including the miR-181a putative binding site, was cloned downstream of the luciferase reporter gene. The mutant form, with a mutated putative miR-181a binding site was also cloned in the same manner. As expected, transfection with the miR-1181a mimic significantly decreased the luciferase activity of the reporter gene containing the wild-type 3’-UTR, whereas transfection with the inhibitor caused the activity to increased (Figure 2B and 2C). Subsequently, we transfected dairy cow hepatocytes with miR-181a mimics. As expected, RT-qPCR analysis revealed that miR-181a was overexpressed (Supplementary Figure 4A), whereas SIRT1 mRNA expression levels were significantly decreased in these hepatocytes compared with control hepatocytes (Figure 2D). Moreover, western blot analysis showed that SIRT1 and PGC-1α protein expression levels were significantly decreased, but acetylated PGC-1α levels were significantly increased in transfected hepatocytes compared with control hepatocytes (Figure 2E). PGC-1α is a direct target of SIRT1, which activates PGC-1α mainly through its ability to deacetylate its coactivator [32]. Thus, PGC-1α protein and acetylation levels represent SIRT1 activities. Besides, hepatocytes transfected with miR-181a inhibitors exhibited significantly decreased miR-181a expression and increased SIRT1 mRNA expression compared with control hepatocytes (Supplementary Figure 4B, Figure 2F). In addition, SIRT1 and PGC-1α protein expression levels were markedly increased, and acetylated PGC-1α levels were significantly decreased in hepatocytes transfected with miR-181a inhibitors compared with control hepatocytes (Figure 2G). Taken together, these data clearly suggest that SIRT1 is a direct target of miR-181a.

More importantly, SIRT1 expression and activity were significantly decreased in the livers of dairy cows and patients with NAFLD, as well as HFD and ob/ob mice, compared with the corresponding control (Figure 2H–2M; Supplementary Figure 5A and 5B). Furthermore, SIRT1 expression and activity were significantly decreased in a dose-dependent manner in NEFA-treated hepatocytes compared with control hepatocytes (Figure 2N and 2O). Taken together, these data indicate that high miR-181a expression may partially reduce SIRT1 expression and activity and miR-181a may be a regulator of glucose and lipid metabolism.

### MiR-181a overexpression impairs and miR-181a inhibition improves glucose and lipid metabolism in hepatocytes

**Figure 3:**
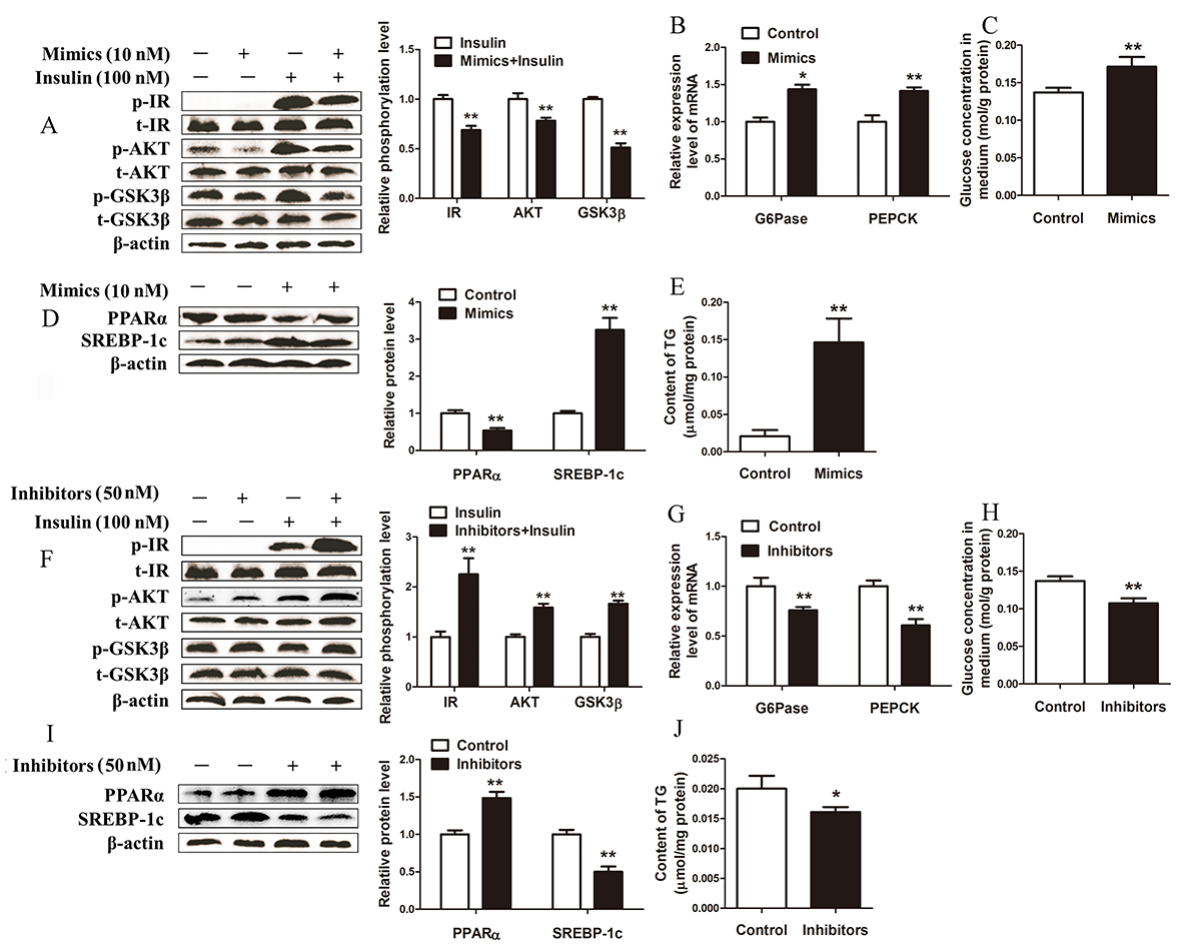
MiR-181a overexpression impairs and miR-181a inhibition improves glucose and lipid metabolism *in vitro* (A, B, C, D and E) Hepatocytes were transfected with 10 *nM* miR-181a mimics or negative controls in the absence or presence of 100 *nM* insulin. **(A)** Immunoblot analysis (left) and quantification (right) of insulin-stimulated phosphorylation of IR, Akt and GSK3β in hepatocytes. **(B)** The mRNA expression levels of *G6Pase* and *PEPCK* in hepatocytes. **(C)** Glucose concentration in medium. **(D)** Immunoblot analysis (left) and quantification (right) of SREBP-1c and PPARα expression in hepatocytes. **(E)** TG content in hepatocytes. (F, G, H, I and J) Hepatocytes were transfected with 50 *nM* miR-181a inhibitors or negative controls in the absence or presence of 100 *nM* insulin. **(F)** Immunoblot analysis (left) and quantification (right) of insulin-stimulated phosphorylation of IR, Akt and GSK3β in hepatocytes. **(G)** The expression levels of *G6Pase* and *PEPCK* in hepatocytes. **(H)** Glucose concentration in medium. **(I)** Immunoblot analysis (left) and quantification (right) of SREBP-1c and PPARα expression in hepatocytes. **(J)** TG content in hepatocytes. ^*^P < 0.05, ^**^P < 0.01. All experiments were repeated at least three times and representative results are shown.

To investigate whether increased miR-181a expression contributes to the development of metabolic disorders, we used miR-181a mimics to overexpress miR-181a in dairy cow hepatocytes. First, we assessed the effects of miR-181a on insulin signaling in hepatocytes stimulated with insulin. The results showed that insulin-stimulated IR, Akt and GSK3β phosphorylation levels were significantly decreased in hepatocytes transfected with miR-181a mimics compared with control hepatocytes (Figure 3A). Consistent with these findings, the mRNA expression levels of the gluconeogenic genes glucose-6-phosphatase (*G6Pase*) and phosphoenolpyruvate carboxykinase (*PEPCK*) were all elevated in hepatocytes overexpressing miR-181a compared with control hepatocytes (Figure 3B). Moreover, due to the effects of decreased insulin sensitivity and increased gluconeogenesis, the glucose level of the medium in the miR-181a-overexpression group was significantly higher than that of the medium in the control group (Figure 3C). We next determined the effects of the miR-181a mimics on lipid metabolism in hepatocytes. We found that overexpressing miR-181a decreased PPARα protein expression levels in the indicated group of hepatocytes compared with the control group of hepatocytes (Figure 3D). Conversely, SREBP-1c protein expression levels were increased in hepatocytes transfected with miR-181a mimics compared with control hepatocytes (Figure 3D). Furthermore, hepatocytes transfected with miR-181a mimics exhibited a significant increase in TG content compared with control hepatocytes (Figure 3E). Taken together, these results demonstrate that miR-181a overexpression impairs insulin sensitivity and glucose and lipid metabolism in the hepatocytes of dairy cows.

The above observations prompted us to evaluate the effects of inhibiting endogenous miR-181a on hepatocyte glucose and lipid metabolism. Consistent with the results of the above experiment, the results of this experiment showed that inhibiting miR-181a in hepatocytes significantly increased insulin-stimulated IR, Akt and GSK3β phosphorylation (Figure 3F) and significantly decreased *G6Pase* and *PEPCK* mRNA expression in the miR-181a inhibitor-treated hepatocyte group compared with the control group (Figure 3G). Moreover, the medium glucose concentration of the indicated group was significantly lower than that of the control group (Figure 3H), and PPARα and SREBP-1c protein expression levels were significantly higher and lower in the miR-181a inhibitor-treated hepatocyte group than the control group, respectively (Figure 3I). Consequently, hepatocyte TG content was significantly decreased in the miR-181a inhibitor-treated hepatocyte group compared with the control group (Figure 3J). Collectively, these data suggest that inhibiting endogenous miR-181a improves insulin sensitivity and glucose and lipid metabolism in hepatocytes.

### Inhibition of miR-181a alleviates high NEFA concentration-induced metabolic disorders by enhancing SIRT1 expression and activity in hepatocytes

**Figure 4:**
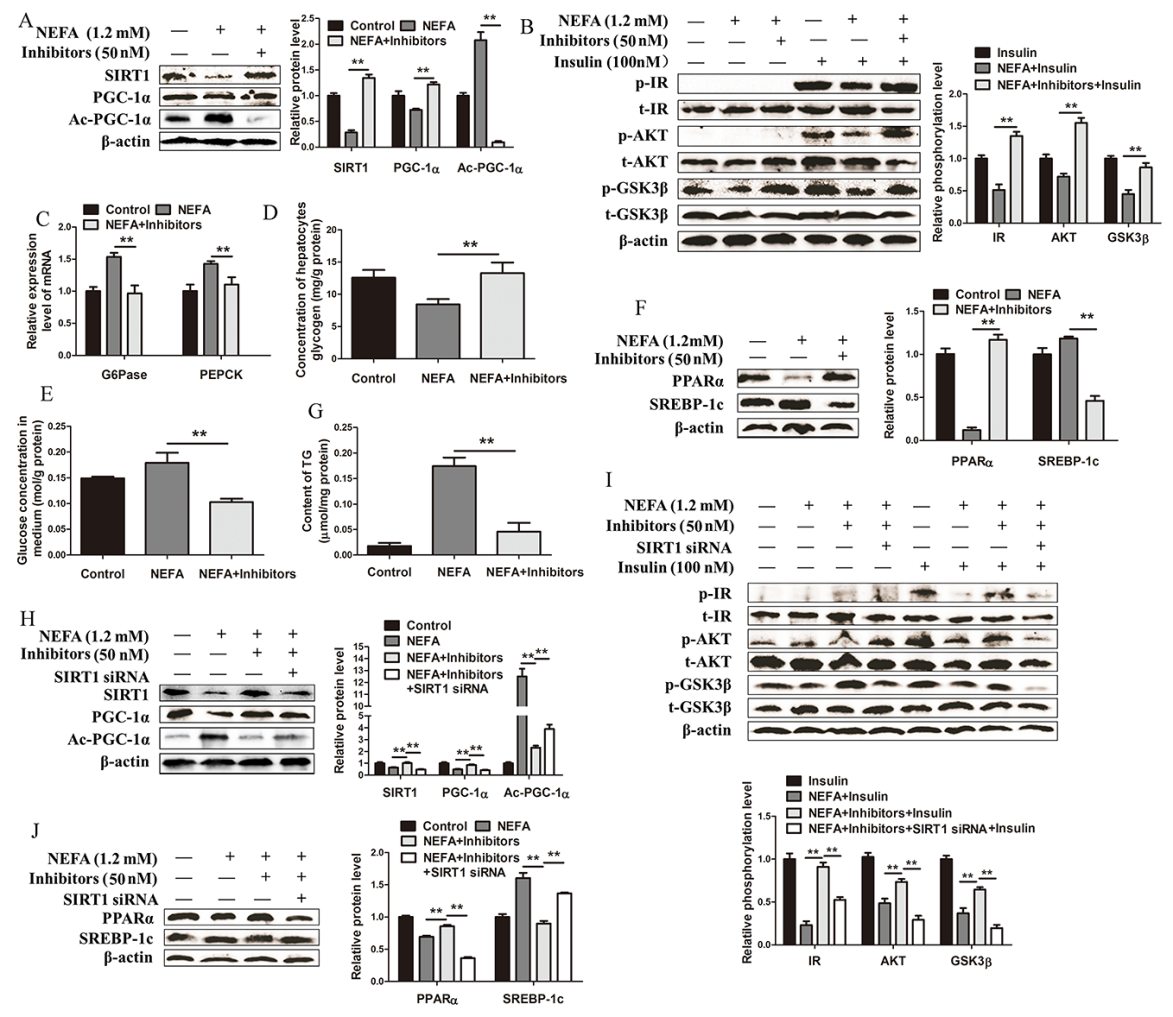
Inhibition of miR-181a alleviates high NEFA concentration-induced metabolic disorder by enhancing SIRT1 expression and activity in hepatocytes **(A, B, C, D, E, F and G)** Hepatocytes were divided into 3 groups as follows: a control group (transfected with 50 *nM* negative control), NEFA group (treated with 1.2 *mM* NEFA), and miR-181a + NEFA group (transfected with 50 *nM* miR-181a inhibitors and then treated with 1.2 *mM* NEFA). B was followed with or without 100 *nM* insulin. **(A)** Immunoblot analysis (left) and quantification (right) of SIRT1, PGC-1α and acetylated PGC-1α in hepatocytes. **(B)** Immunoblotting analysis (left) and quantification (right) of insulin-stimulated phosphorylation of IR, AKT and GSK3β in hepatocytes. **(C)** The expression levels of *G6Pase* and *PEPCK* in hepatocytes. **(D)** Glycogen content in hepatocytes. **(E)** Glucose concentration in medium. **(F)** Immunoblot analysis (left) and quantification (right) of SREBP-1c and PPARα in hepatocytes. **(G)** TG content in hepatocytes. (H, I and J) Hepatocytes were divided into 4 groups as follows: a control group (transfected with 50 *nM* negative control), NEFA group (treated with 1.2 *mM* NEFA), and miR-181a + NEFA group (transfected with 50 *nM* miR-181a inhibitors and then treated with 1.2 *mM* NEFA), miR-181a + NEFA + SIRT1 siRNA group (transfected with 50 *nM* miR-181a inhibitors and SIRT1 siRNA then treated with 1.2 *mM* NEFA). I was followed with or without 100 *nM* insulin. **(H)** Immunoblot analysis (left) and quantification (right) of SIRT1, PGC-1α and acetylated PGC-1α in hepatocytes. **(I)** Immunoblotting analysis (top) and quantification (bottom) of insulin-stimulated phosphorylation of IR, AKT and GSK3β in hepatocytes. **(J)** Immunoblotting analysis (left) and quantification (right) of SREBP-1c and PPARα in hepatocytes. ^*^P < 0.05, ^**^P < 0.01. All experiments were repeated at least three times and representative results are shown.

Based on the above findings, we hypothesized that inhibiting miR-181a may improve insulin sensitivity and decrease lipid accumulation under NEFA treatment conditions in hepatocytes. Hepatocytes co-treated with NEFA (1.2 *mM*) and miR-181a inhibitors (50 *nM*) displayed a significant decrease in their miR-181a expression level compared with hepatocytes treated with NEFA alone (Supplementary Figure 6). The abovementioned decreases in SIRT1 protein expression levels and activity in hepatocytes were also significantly enhanced by treatment with miR-181a inhibitors (Figure 4A). As expected, the miR-181a inhibitors markedly increased insulin-induced IR, Akt and GSK3β phosphorylation in the indicated group of hepatocytes compared with the group of hepatocytes treated with NEFA alone (Figure 4B). In addition, NEFA-induced *G6Pase* and *PEPCK* mRNA expression levels were significantly reduced by the miR-181a inhibitors (Figure 4C). Moreover, the glycogen content in hepatocytes and the glucose level in the medium were significantly higher and lower in hepatocytes co-treated with NEFA and miR-181a inhibitors than hepatocytes treated with NEFA alone, respectively (Figure 4D and 4E). Taken together, these data indicate that miR-181a inhibitors improve glucose homeostasis in NEFA-stimulated hepatocytes.

Furthermore, we also evaluated lipid metabolism in hepatocytes co-treated with NEFA and miR-181a inhibitors. The results showed that hepatocytes co-treated with NEFA and miR-181a inhibitors displayed a significant increase in their PPARα protein expression levels and a decrease in their SREBP-1c protein expression levels compared with hepatocytes treated with NEFA alone (Figure 4F). In addition, we found that inhibiting miR-181a reversed the effects of NEFA on the lipogenic genes fatty acid synthase (*FAS*) and stearoyl-CoA desaturase 1 (*SCD-1*) and the lipolytic genes carnitine palmitoyltransferase I (*CPT-I)* and carnitine palmitoyltransferase II (*CPT-II*) (Supplementary Figure 7). Consistent with these findings, we found that NEFA-induced TG content in hepatocytes was significantly reduced by miR-181a inhibitors (Figure 4G). Taken together, these results demonstrate that inhibiting miR-181a is sufficient for preventing NEFA-induced abnormal lipid metabolism in hepatocytes.

To determine whether SIRT1 mediates the effects of miR-181a on hepatocyte glucose and lipid metabolism, we transfected hepatocytes with SIRT1 siRNA, which decreased SIRTI and PGC-1α protein expression and increased PGC-1α acetylation levels, changes indicative of SIRT1 inhibition (Supplementary Figure 8). Hepatocytes co-treated with NEFA and miR-181a inhibitors displayed a significant increase in SIRT1 protein expression levels and activity compared with cells treated with NEFA alone, changes that were reversed by SIRT1 siRNA transfection (Figure 4H). Moreover, insulin-dependent IR, Akt and GSK3β phosphorylation was significantly increased by miR-181a inhibition under NEFA treatment conditions, this effect was reversed by SIRT1 siRNA (Figure 4I). In addition, miR-181a inhibitor-induced increases in PPARα expression and decreases in SREBP-1c expression were also blocked by SIRT1 siRNA transfection (Figure 4J). Based on these findings, we concluded that SIRT1 mediates the regulatory function of miR-181a in glucose and lipid metabolism.

### SIRT1 knockdown impairs and SIRT1 overexpression improves glucose and lipid metabolism in hepatocytes

**Figure 5:**
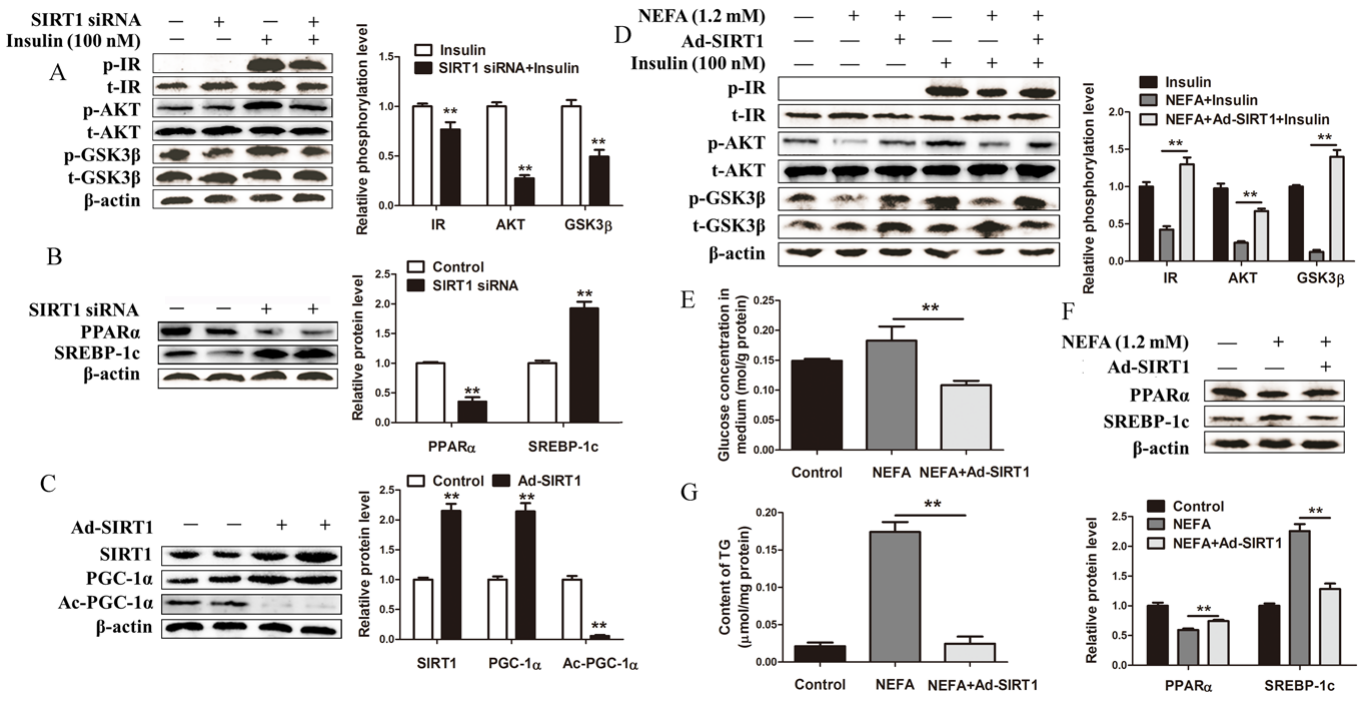
SIRT1 knockdown impairs and SIRT1 overexpression improves glucose and lipid metabolism in hepatocytes **(A)** Immunoblot analysis (left) and quantification (right) of insulin-stimulated phosphorylation of IR, Akt and GSK3β protein levels in hepatocytes. Hepatocytes were transfected with SIRT1 siRNA with or without 100 *nM* insulin. **(B)** Immunoblot analysis (left) and quantification (right) of SREBP-1c and PPARα protein levels in hepatocytes. Hepatocytes were transfected with SIRT1 siRNA. **(C)** Immunoblot analysis (left) and quantification (right) of SIRT1, PGC-1α and acetylated PGC-1α in hepatocytes. Hepatocytes were infected with Ad-SIRT1. **(D)** Immunoblotting analysis (left) and quantification (right) of insulin-stimulated phosphorylation of IR, AKT and GSK3β in hepatocytes. Hepatocytes were treated with 1.2 *mM* NEFA or 1.2 *mM* NEFA and Ad-SIRT1 with or without 100 *nM* insulin. (E, F and G) Hepatocytes were treated with 1.2 *mM* NEFA or 1.2 *mM* NEFA and Ad-SIRT1. **(E)** Glucose concentration in medium. **(F)** Immunoblot analysis (top) and quantification (bottom) of SREBP-1c and PPARα in hepatocytes. **(G)** TG contents in hepatocytes. ^*^P < 0.05, ^**^P < 0.01. All experiments were repeated at least three times and representative results are shown.

To directly investigate the role of SIRT1 in control of glucose and lipid metabolism in dairy cow hepatocytes, we silenced or overexpressed SIRT1 with siRNA or adenoviruses, respectively. We first examined the effect of SIRT1 siRNA on the insulin signaling pathway. Consistent with the results of the experiments involving the miR-181a mimics, the results of this experiment showed that insulin-stimulated IR, Akt and GSK3β phosphorylation was significantly decreased in hepatocytes transfected with SIRT1 siRNA compared with control hepatocytes (Figure 5A). Furthermore, transfection with SIRT1 siRNA significantly decreased PPARα protein expression levels and increased SREBP-1c protein expression levels in hepatocytes (Figure 5B). These results indicate that SIRT1 inhibition impairs glucose and lipid metabolism in hepatocytes.

We also assessed the effects of Ad-SIRT1 on glucose and lipid metabolism in hepatocytes. Western blot analysis showed that hepatocytes infected with Ad-SIRT1 displayed increased SIRT1 and PGC-1α protein expression levels and reduced PGC-1α acetylation levels, findings indicative of increases in SIRT1 expression and activity, compared with control hepatocytes (Figure 5C). In addition, we found that infection with Ad-SIRT1 prevented NEFA-mediated insulin signaling pathway impairment (Figure 5D). Glucose concentration in medium was also significantly decreased in hepatocytes co-treated with NEFA and Ad-SIRT1 compared with hepatocytes treated with NEFA alone (Figure 5E). We next investigated lipid metabolism in hepatocytes co-treated with NEFA and Ad-SIRT1 and found that NEFA treatment significantly decreased PPARα protein expression levels and increased SREBP-1c protein expression levels in treated hepatocytes compared with control hepatocytes, changes that were reversed by Ad-SIRT1 (Figure 5F). In addition, Ad-SIRT1 infection significantly decreased hepatocyte TG content under NEFA treatment conditions (Figure 5G). Taken together, these data suggest that SIRT1 overexpression improves NEFA-induced glucose and lipid metabolic disorders in hepatocytes.

### MiR-181a overexpression impairs and miR-181a inhibition improves glucose and lipid homeostasis in HepG2 cells

**Figure 6:**
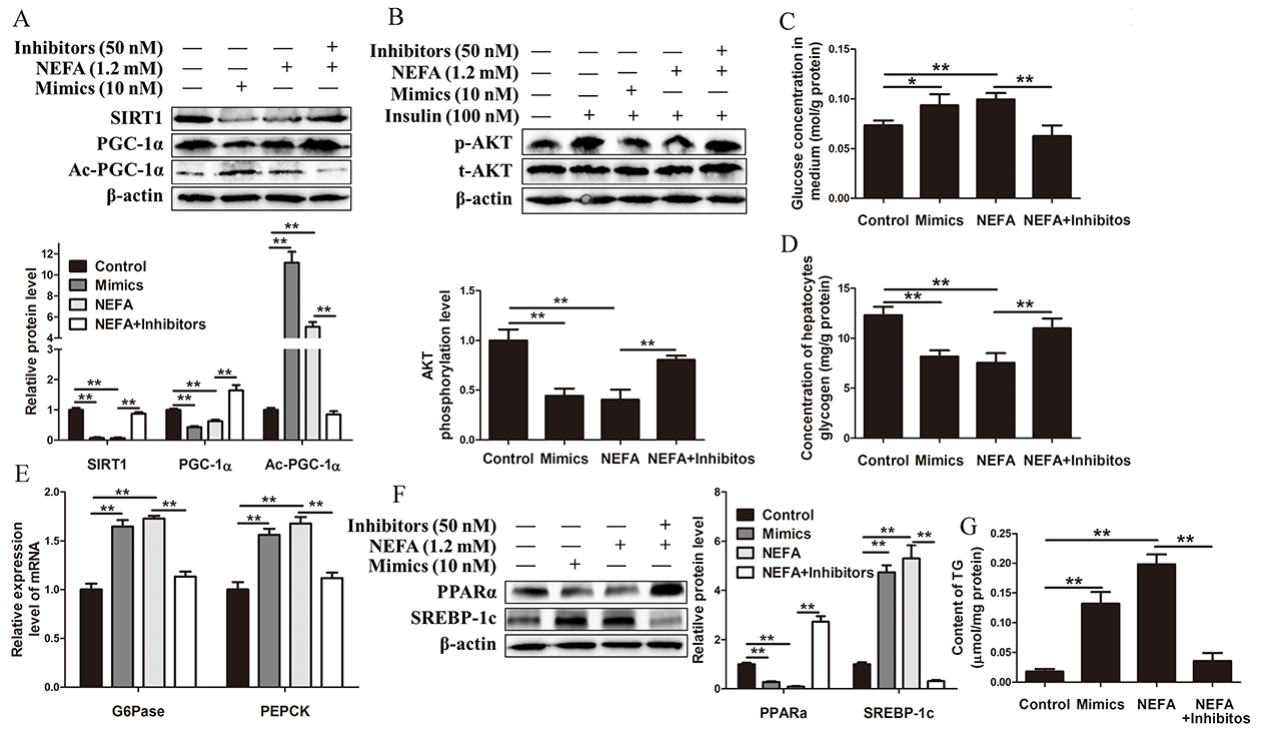
MiR-181a overexpression impairs and miR-181a inhibition improves glucose and lipid homeostasis in HepG2 cells HepG2 cells were divided into 4 groups as follows: a control group, mimics group (HepG2 cells transfected with 10 *nM* mimics), NEFA group (treated with 1.2 *mM* NEFA), and miR-181a + NEFA group (transfected with 50 *nM* miR-181a inhibitors and then treated with 1.2 *mM* NEFA). B was followed with or without 100 *nM* insulin. **(A)** Immunoblot analysis (top) and quantification (bottom) of SIRT1, PGC-1α and acetylated PGC-1α in HepG2 cells. **(B)** Immunoblotting analysis (top) and quantification (bottom) of insulin-stimulated phosphorylation of AKT in HepG2 cells. **(C)** Glucose concentration in medium. **(D)** The glycogen content in HepG2 cells. **(E)** The mRNA expression levels of *G6Pase* and *PEPCK*. **(F)** Immunoblot analysis (left) and quantification (right) of SREBP-1c and PPARα in HepG2 cells. **(G)** TG content in HepG2 cells. ^*^P < 0.05, ^**^P < 0.01. All experiments were repeated at least three times and representative results are shown.

We also investigated the effects of miR-181a-SIRT1 on glucose and lipid metabolism in HepG2 cells. The results showed that HepG2 cells transfected with miR-181a mimics or treated with NEFA displayed decreases in SIRTI and PGC-1α protein expression levels and increases in PGC-1α acetylation levels, indicating inhibited SIRT1 expression and activity (Figure 6A). These effects were reversed in HepG2 cells transfected with miR-181a inhibitors (Figure 6A). In addition, AKT phosphorylation levels and glycogen content were significantly decreased in HepG2 cells transfected with miR-181a mimics or treated with NEFA compared with control cells (Figure 6B and 6D), and the medium glucose concentration and *G6Pase* and *PEPCK* mRNA expression levels were significantly higher in HepG2 cells transfected with miR-181a mimics or treated with NEFA than in control cells (Figure 6C and 6E). These data indicate that insulin sensitivity and glucose metabolism are impaired by NEFA or miR-181a mimics in HepG2 cells. More importantly, inhibition of miR-181a significantly improved insulin sensitivity and attenuated the impairments in glucose metabolism induced by NEFA in HepG2 cells (Figure 6B–6E).

In addition, HepG2 cells transfected with miR-181a mimics or treated with NEFA also displayed decreased PPARα protein expression levels and increased SREBP-1c protein expression levels and TG content compared with control cells (Figure 6F and 6G). As expected, inhibition of miR-181a reversed the effects of NEFA on lipid metabolism in HepG2 cells (Figure 6F and 6G). These data indicate that miR-181a overexpression impairs and miR-181a inhibition improves glucose and lipid homeostasis in HepG2 cells.

### MiR-181a overexpression or SIRT1 knockdown impairs glucose and lipid metabolism *in vivo*

**Figure 7:**
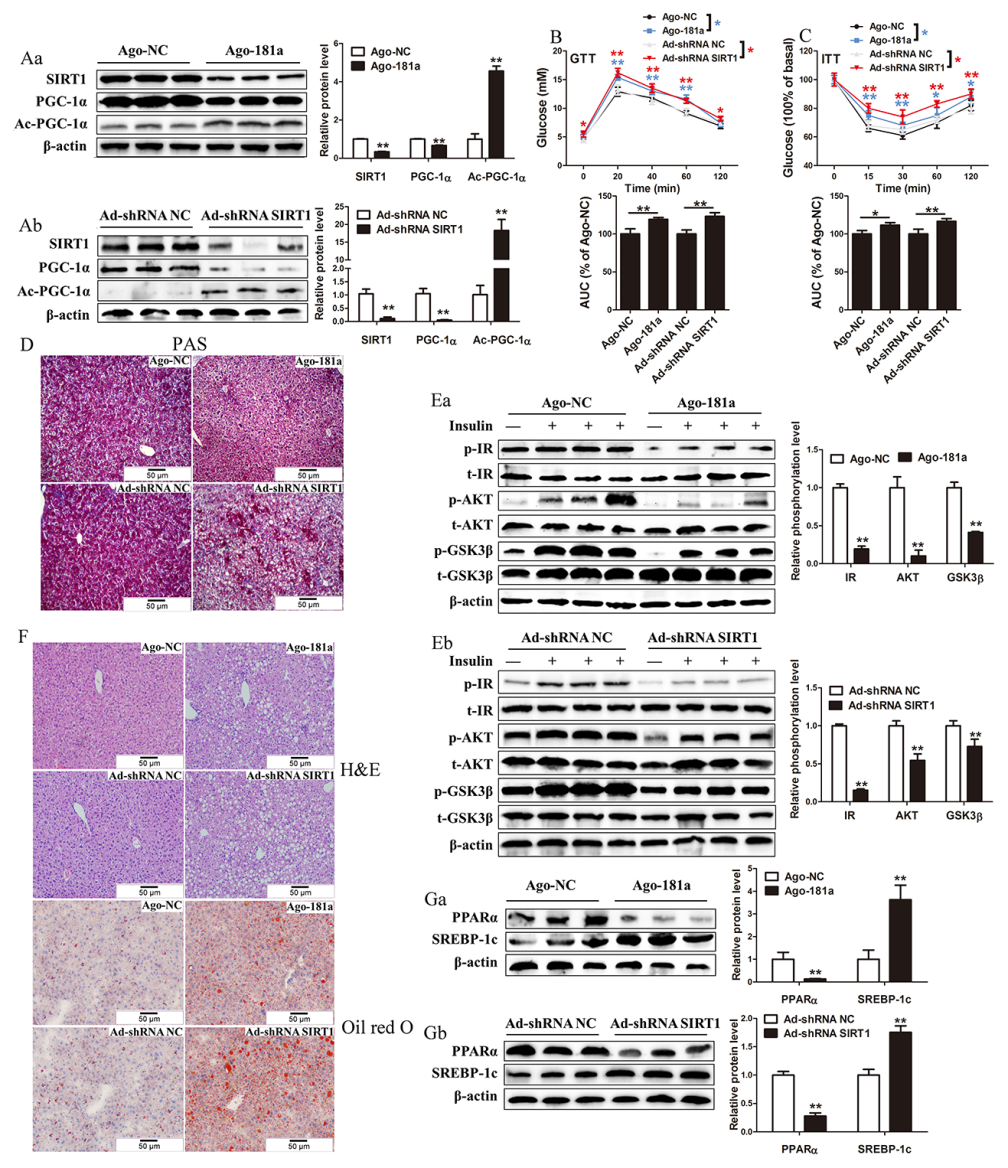
MiR-181a overexpression or SIRT1 knockdown impairs glucose and lipid metabolism *in vivo* **(Aa and Ab)** Immunoblotting analysis (left) and quantification (right) of SIRT1, PGC-1α and acetylated PGC-1α in the liver of mice injected with Ago-181a or Ago-NC or Ad-shRNA NC or Ad-shRNA SIRT1. **(B)** Glucose tolerance test of mice injected with Ago-181a (*n* = 7) or Ago-NC (*n* = 7) or Ad-shRNA NC (*n* = 6) or Ad-shRNA SIRT1 (*n* = 6). **(C)** Insulin tolerance test of mice injected with Ago-181a (*n* = 7) or Ago-NC (*n* = 7) or Ad-shRNA NC (*n* = 6) or Ad-shRNA SIRT1 (*n* = 6). **(D)** Representative images of PAS staining (original magnification × 20) of liver sections from mice injected with Ago-181a or Ago-NC or Ad-shRNA NC or Ad-shRNA SIRT1. **(Ea and Eb)** Immunoblotting analysis (top) and quantification (bottom) of insulin-stimulated phosphorylation of IR, AKT and GSK3β in liver of mice injected with Ago-181a or Ago-NC or Ad-shRNA NC or Ad-shRNA SIRT1. **(F)** Representative images of H&E staining (left, original magnification × 20) and Oil-red staining (right, original magnification × 20) of liver sections from mice injected with Ago-181a or Ago-NC or Ad-shRNA NC or Ad-shRNA SIRT1. **(Ga and Gb)** Immunoblotting analysis (top) and quantification (bottom) of SREBP-1c and PPARα in the liver of mice injected with Ago-181a or Ago-NC or Ad-shRNA NC or Ad-shRNA SIRT1. ^*^P < 0.05, ^**^P < 0.01. All experiments were repeated at least three times and representative results are shown.

To explore the function of miR-181a and SIRT1 *in vivo*, we used Antagomir of miR-181 (Ago-181a) and adenovirus expressing a mouse SIRT1 short hairpin RNA (shRNA) (Ad-shRNA SIRT1) to overexpress miR-181a and knock down SIRT1, respectively, in the livers of mice. MiR-181a expression in the liver of Ago-181a-treated mice was 32-fold higher than that in the livers of control mice (Supplementary Figure 9A). In addition, hepatic SIRT1 protein expression levels and activity were significantly decreased in these mice compared with control mice (Figure 7Aa). Moreover, SIRT1 knockdown significantly decreased hepatic SIRT1 protein expression levels and activity in the indicated group of mice compared with the control group of mice (Figure 7Ab). Hepatic overexpression of miR-181a or knockdown of SIRT1 both resulted in augmented random glucose levels (Supplementary Figure 9B), and the glucose-tolerance tests (GTT) and insulin-tolerance tests (ITT) results showed that glucose and insulin sensitivity were impaired in mice injected with Ago-181a or Ad-shRNA SIRT1 compared with control mice (Figure 7B and 7C). We also measured glycogen content in the livers of mice in which miR-181a was overexpressed or SIRT1 was silenced. Periodic acid-schiff (PAS) staining showed that glycogen content was reduced in these livers compared with control livers (Figure 7D). Consistent with this result, the results of the glycogen content analysis suggested that glycogen levels were significantly decreased after Ago-181a or Ad-shRNA SIRT1 injection in the indicated groups compared with the control group (Supplementary Figure 9C). Moreover, insulin-induced IR, Akt and GSK3β phosphorylation was decreased in the livers of mice after Ago-181a or Ad-shRNA SIRT1 injection, findings indicative of impaired hepatic insulin signaling (Figure 7Ea and 7Eb). Collectively, these findings indicate that overexpressing miR-181a or knocking down SIRT1 in mice causes insulin resistance and impairs glucose homeostasis.

We next assessed the functional contribution of upregulated miR-181a or silenced SIRT1 to lipid metabolism. We found that hepatic TG content was significantly higher in mice injected with Ago-181a or Ad-shRNA SIRT1 than in control mice (Supplementary Figure 9D). The increases in lipid accumulation were confirmed by H&E and Oil Red O staining (Figure 7F). Consistent with these results, our results also showed that PPARα protein expression was decreased, while SREBP-1c protein expression was increased in mice in which miR-181a was overexpressed or SIRT1 was silenced compared with control mice (Figure 7Ga and 7Gb). These data strongly suggest that overexpressing miR-181a or knocking down SIRT1 increases lipid accumulation *in vivo*.

## DISCUSSION

Insulin resistance and NAFLD are often associated with a cluster of other metabolic abnormalities, including dyslipidemia, hyperinsulinemia and hypertension, collectively termed the “metabolic syndrome” [3, 33–35]. The pathogenesis of this disease is believed to comprise several steps, beginning with excess lipid accumulation in non-adipose tissue due to increased exportation of free fatty acids from adipose tissue [36]. There are many different diet-induced and genetic animal models of NAFLD, and each has its own advantages and disadvantages. However, dairy cows, which present with naturally occurring NAFLD during transition periods, have never been studied. The hyperlipidemia, hyperglycemia and hyperinsulinemia observed in dairy cows generally occur earlier in lactation [37, 38]. The disturbances associated with NAFLD in cows have many similarities with those associated with type 2 diabetes and NAFLD in humans. In our experiments, we found that hyperlipidemia, hyperglycemia and hyperinsulinemia developed in patients and dairy cows with NAFLD. Furthermore, we found that insulin signaling was impaired and that lipid accumulation was increased in the livers of NAFLD dairy cows compared with those of control dairy cows. These data strongly indicated that disorders of glucose and lipid metabolism and insulin resistance are present in dairy cows and patients with NAFLD. Thus, dairy cows with NAFLD may be an ideal model for investigating the mechanism underlying NAFLD development and progression. In addition, dairy cows can provide researchers with more hepatocytes than mice or rats, which facilitates the performance of cellular experiments.

It has been well recognized that miRNAs play important roles in hepatic function and energy metabolism [39]. For example, it has been shown that global or liver-specific overexpression of miR-26a prevented obesity-induced metabolic complications by targeting several key regulators of hepatic energy metabolism and insulin signaling in mice fed an HFD [40]. MiR-122, the most abundant miRNA in the liver, plays a key role in the epigenetic regulation of the expression of genes related to cholesterol and lipid metabolism [41, 42]. These studies demonstrate that miRNAs play a crucial role in hepatic glucose and lipid metabolism. In addition, several studies have shown that liver miRNA expression is dysregulated in postpartum dairy cows [43, 44].

MiR-181a plays crucial roles in tumorigenesis by targeting critical regulators of metastasis, cell cycle, and differentiation [27, 45, 46]. In addition, miR-181a acts as a potent tumor suppressor in the liver [47], the central organ involved in controlling glucose homeostasis and lipid metabolism. Despite the significance of miR-181a in cancer, its function in metabolism and metabolic disease is poorly characterized. In our study, we were surprised to find that miR-181a expression levels were significantly increased in the liver and blood of patients and dairy cows with NAFLD, as well as in HFD and ob/ob mice and hepatocytes treated with NEFA, compared with the corresponding controls. Thus, we hypothesized that alterations in miR-181a expression may affect hepatic energy metabolism. Subsequent experiments showed that miR-181a overexpression impaired the insulin signaling pathway and glucose metabolism, elevated SREBP-1c protein expression levels and TG contents, and decreased PPARα protein expression levels in dairy cow hepatocytes and HepG2 cells. In contrast, miR-181a inhibition abolished NEFA-induced metabolic disorders in dairy cow hepatocytes and HepG2 cells. More importantly, overexpressing miR-181a in mouse livers reduced glucose and insulin sensitivity and hepatic glycogen content and increased hepatic lipid accumulation in treated livers compared with control livers. Taken together, our data showed that miR-181a is a negative regulator of hepatic glucose and lipid metabolism. However, a previous study showed that miR-181a suppressed lipogenesis in bovine mammary epithelial cells by targeting acyl-CoA synthetase long-chain family member 1 (ACSL1) [48], and another study showed that miR-181a inhibited lipid accumulation by targeting isocitrate dehydrogenase 1 (IDH1) in mouse embryonic fibroblast cells [49]. The discrepancies between the results of the above studies and those of our study may be related mainly to differences in cell types, targets and genetic backgrounds or some combination thereof.

A recent study reported that miR-181a and b, miR-199b, miR-135a and miR-205 targeted endogenous SIRT1 and downregulated its expression in mouse embryonic stem cells [28]. Our results implied that miR-181a overexpression decreased SIRT1 expression and activity and subsequently decreased PGC-1α protein expression levels and increased PGC-1α acetylation levels in cow hepatocytes, HepG2 cells and mice compared with control cells and mice. Furthermore, miR-181a expression levels were increased and SIRT1-PGC-1α pathway activity was decreased in the liver of dairy cows and patients with NAFLD, as well as in HFD mice and ob/ob mice and hepatocytes treated with NEFA, compared with the corresponding controls. We also found that miR-181a inhibition rescued NEFA-mediated SIRT1-PGC-1α pathway inhibition. These data demonstrated that SIRT1 was a direct target of miR-181a.

SIRT1 is an essential regulator of systemic energy homeostasis. A previous study showed that hepatic deletion of SIRT1 in mice causes hepatic glucose overproduction, chronic hyperglycemia and oxidative stress [50]. Conversely, Zang and colleagues showed that adenovirus-mediated hepatic overexpression of SIRT1 attenuates insulin resistance, restores glucose homeostasis, and ameliorates hepatic steatosis [51]. Given the results of these studies, we investigated the function of hepatic SIRT1 in glucose and lipid metabolism. Our data showed that SIRT1 overexpression in hepatocytes attenuated NEFA-induced insulin resistance, glucose metabolic derangements and lipid accumulation. In addition, SIRT1 knockdown in mice and cow hepatocytes impaired glucose and insulin sensitivity, decreased glycogen content, and increased lipid accumulation. Taken together, these data demonstrate that SIRT1 serves as a positive regulator of glucose and lipid metabolism *in vivo* and *in vitro*.

**Figure 8:**
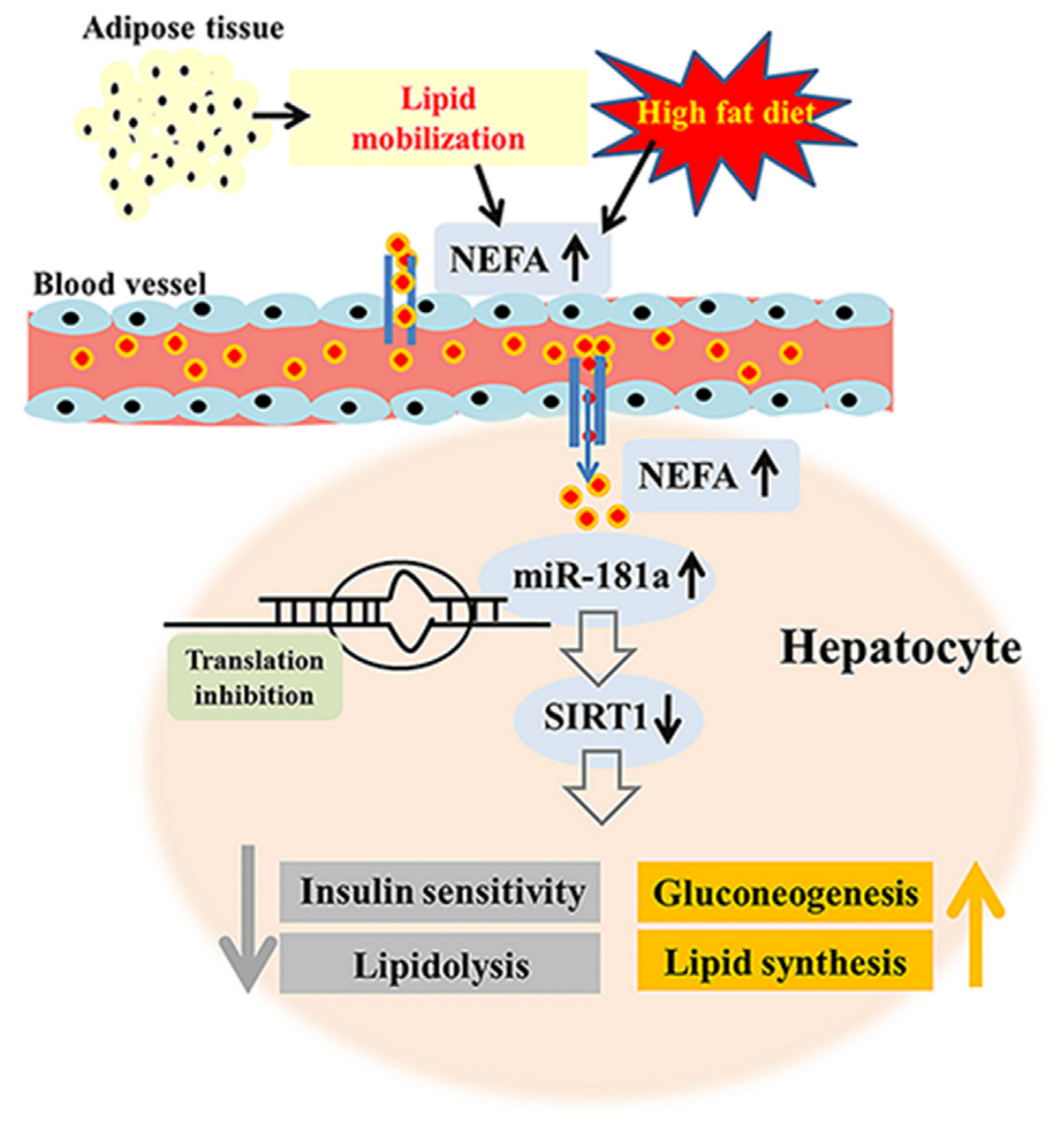
The proposed model for the metabolic regulation of miR-181a-SIRT1 Excess NEFA in the liver increases miR-181a expression, which in turn decreases SIRT1 expression and activity via translation inhibition, resulting in reduced lipidolysis, impaired insulin sensitivity, and increased gluconeogenesis and lipid synthesis. Therefore, inhibiting miR-181a or increasing SIRT1 expression and activity may contribute to reductions in metabolic dysfunction in dairy cows or patients with NAFLD.

The present study demonstrated that excess NEFA increases miR-181a expression, which in turn decreases SIRT1 expression and activity in hepatocytes. SIRT1 downregulation subsequently decreases lipidolysis, impairs insulin sensitivity, and increases gluconeogenesis and lipid synthesis (Figure 8). These findings are indicative of the importance of hepatic miR-181a-SIRT1 in glucose and lipid metabolism and suggest that miR-181a-SIRT1 has promise as a novel target for the treatment of NAFLD. Furthermore, our data also demonstrated that cows with NAFLD may be an ideal animal model for the study of human NAFLD.

## MATERIALS AND METHODS

### Reagents and antibodies

Oleic acid, linoleic acid, palmitic acid, stearic acid and palmitoleic acid were obtained from Sigma (St. Louis, MO, USA). The mimics, inhibitors and Ago-181a or Ago-NC were purchased from RiboBio (RiboBio Co. Ltd, Guangzhou, China). X-tremeGENE siRNA Transfection Reagent was from Roche (Mannheim, Germany). The mammalian cell protein extraction kit was from Beyotime (Jiangsu, China). The antibodies against acety-lysine (Cat: 9441), tyr1150/1151-phosphorylated IR (Cat: 3024; 95 kDa), Akt (Cat: 9272; 60 kDa), Ser473-phosphorylated Akt (Cat: 4060; 60 kDa) and Ser9-phosphorylated (GSK3β) (Cat: 9336; 46 kDa) were purchased from Cell Signaling Technology(Berverly, MA, USA), antibody against SREBP-1c (Cat: NB100-2215; 65 kDa) (Novus Biologicals, Littleton, CO, USA), antibodies against β-actin (Cat: ab8226; 42 kDa), PPARα (Cat: ab8934; 52 kDa), IR (Cat: ab69508; 95kDa) and GSK3β (Cat: ab69739; 47 kDa) were purchased from Abcam (Cambridge, England), antibodies against SIRT1 (Cat: sc-15404; 120 kDa) and PGC-1α (Cat: sc13067; 90 kDa) were purchased from Santa Cruz Biotechnology (Santa Cruz, CA, USA). Bovine SIRT1 small interference RNA (siRNA) or control, adenovirus expressing bovine SIRT1 (Ad-SIRT1), Ad-shRNA SIRT1 or negative control shRNA (Ad-shRNA NC) were purchased from Hanbio (Shanghai, China)

### Animals

The Ethics Committee on the Use and Care of Animals at Jilin University approved the study protocol (Changchun, China).

Six-week-old male C57BL/6J and ob/ob mice were purchased from the Laboratory Animal Center of Jilin University. All the mice were maintained in a specific pathogen-free facility under a 12-h dark/light cycle (lights on at 06:30 h), a temperature of 23 ± 3 °C and a humidity of 35 ± 5%.

For the HFD-induced obesity studies, the C57BL/6J mice were fed control diet (10% kcal in fat; D12450B, Research diet, New Brunswick, NJ, USA) or an HFD (60% kcal in fat; Research diets, D124492) for 16 weeks, and the ob/ob mice were fed control diet (D12450B) for 8 weeks.

The C57BL/6J mice were injected via the tail vein with either Ago-NC or Ago-181a at a dose of 80 mg/kg body weight in 0.2 mL of saline twice a week for 3 consecutive weeks and then used for experiments. Additional C57BL6J mice were injected via the tail vein with either Ad-shRNA SIRT1 or Ad-shRNA NC at a dose of 1 × 10^9^ plaque-forming units (PFU)/mouse. After 12 days, these mice were used for experiments.

To detect insulin signaling *in vivo*, we fasted the mice for 16 h and then intraperitoneally injected them with PBS or human insulin (Novo Nordisk, Princeton, NJ, USA) at a dose of 0.75 U/kg. Fifteen minutes later, the mice were sacrificed, and livers were collected to measure the expression levels of the proteins involved in insulin signaling by immunoblotting assay.

We chose the lactating Holstein cows with similar number of lactation (median = 3, range = 2 to 4) and days in milk (median = 6 d, range = 3 to 9 d) from a 10,000-cow dairy farm located in Changchun City, Jilin Province, China. Basic descriptions of the cows with NAFLD and the healthy cows are shown in Supplementary Table 2. Hepatic samples were collected by an experienced veterinarian using a liver puncture needle, which was placed between the cows’ 10th and 11th ribs [52]. Blood samples were collected from each cow via coccygeal venipuncture and immediately centrifuged at 3,500 *g* for 15 min at 4 °C. Serum was obtained and stored at -80 °C until analysis.

### Clinical samples

The study comprised 25 obese patients with NAFLD. These patients were selected for bariatric surgery as a treatment. We further studied 15 healthy lean volunteers undergoing selective abdominal surgery such as herniotomy or cholecystectomy. All surgical liver biopsy were collected during surgery. Some of samples were fixed with 10% formalin for H&E staining and other tissues for molecular studies were immediately frozen in liquid nitrogen and stored at -80 °C. None of the patients tested positive for hepatitis B virus (HBV), hepatitis C virus (HCV) and human immunodeficiency virus (HIV) infections. Biopsy with necroinflammation and fibrosis were excluded. Patients with other causes of chronic liver disease or those receiving potentially hepatotoxic drugs were excluded. In addition, patients consumed less than 20 g of alcohol per day. After a 12-h overnight, clinical and anthropometric data, as well as venous blood samples of each patient were obtained. Basic description of patients was presented in Supplementary Table 1. This study was performed in agreement with the Declaration of Helsinki, and with local and national laws. The study protocol was approved by the ethics committee of First Hospital of Jilin University (Changchun, China). Informed consent was obtained from all patients involved in this study.

### Serum sample analysis

Glucose, NEFA, insulin, TG, AST, ALT and γ-GT were measured using biochemical or ELISA kits (Beijing Strong Biotechnologies, Inc., Beijing, China; R&D Systems, Minneapolis, USA) according to the supplier’s protocol, respectively. HbA_1c_ levels were determined using high performance liquid chromatography. HOMA-IR index was calculated according to the formula: [fasting glucose levels (*mM*)] × [fasting serum insulin (mU/L)]/22.5. BMI was calculated according to the formula: [weight (kg)]/ [height (m) ^*^ height (m)].

### Glucose and insulin tolerance tests

GTT was carried out on mice that have been fasted overnight for 16 h. After determination of fasted blood glucose level, each mouse was received an intraperitoneal injection of 2 g/Kg body weight of glucose. Blood glucose levels were detected from tail vein after 20, 40, 60 and 120 min. ITT were carried out in random-fed mice. After measuring basal blood glucose level, each mouse was treated with 0.75 U/Kg body weight of insulin. Blood glucose level was recorded after 15, 30, 60 and 120 min.

### Histology

Liver-tissue samples were fixed in 10% buffered formalin at 4 °C overnight and embedded in paraffin wax. Paraffin sections (5μm) were cut and mounted on glass slides for H&E staining. Cryosections of livers were stained by oil-red and counterstained with hematoxylin. For PAS staining, the livers were embedded in OCT compound. The frozen-embedded specimens were cut at 5 μm thickness and stained for PAS staining.

### Cell culture, treatment and transfection

Dairy cow primary hepatocytes were isolated using the collagenase IV perfusion method and cultured as previously described [53, 54]. The major function of solution A was clear blood off liver tissues and chelate calcium ions. The major function of solution B was to activate collagenase IV. The cell density was adjusted to 1.5×10^6^ cells/mL using adherent medium [RPMI-1640 basic medium (glucose: 2000 mg/L) supplemented with 10% fetal bovine serum, 10^-6^ mol/L insulin, 10^-6^ mol/L dexamethasone, and 10 μg/mL vitamin C], and the hepatocytes were seeded into a 6-well tissue culture plate (2 mL per well). Subsequently, the cells were incubated at 37 °C in 5% CO_2_. After 5 h, the adherent medium was replaced with growth medium (RPMI-1640 basic medium supplemented with 10% fetal bovine serum).

HepG2 cells were purchased from American Type Culture Collection (Rockville, MD, USA) and were routinely cultured in Dulbecco’s modified Eagle medium (DMEM; glucose: 1000 mg/L) supplemented with 10% fetal bovine serum at 37 °C in 5% CO_2_.

Hepatocytes were serum-starved overnight before they were treated. The concentrations of the NEFAs used in this study were chosen based on the serum concentrations of NEFAs in patients and dairy cows with NAFLD [55, 56]. The stock NEFA (52.7 *mM*) solution included oleic acid (22.9 *mM*), linoleic acid (2.6 *mM*), palmitic acid (16.8 *mM*), stearic acid (7.6 *mM*) and paltoleic acid (2.8 *mM*). Hepatocytes were maintained in RPMI-1640 basic medium or DMEM containing 2% bovine serum albumin and treated with 0, 0.6, 1.2 or 2.4 *mM* NEFA for 12 h. To simulate the insulin signaling pathway, we treated the hepatocytes with 100 *nM* insulin for 30 min. To inhibit or increase miR-181a expression, we transfected the hepatocytes with either 10 *nM* miR-181a mimics or 50 *nM* inhibitors for 48 h using X-tremeGENE siRNA Transfection Reagent (Roche), according to the manufacturer’s instructions. To silence SIRT1, we transfected the hepatocytes with SIRT1 siRNA for 48 h using X-tremeGENE siRNA Transfection Reagent (Roche). To overexpress bovine SIRT1 in dairy cow hepatocytes, we infected the cells with Ad-SIRT1 or control vector for 48 h. The cell treatments and detailed groupings are shown in the figure legends.

### Total RNA isolation and real-time quantitative PCR (RT-qPCR)

Serum total RNA was extracted using a miRNeasy Serum/Plasma Kit (QIAGEN, Hilden, Germany), and 30 fmol of synthetic *C. elegans* miR-39-3p (Ribobio) was added to each sample. Total RNA was extracted from the liver tissue samples or hepatocytes with TRIzol reagent (Invitrogen, Carlsbad, CA), according to the manufacturer’s instructions, and cDNA was synthesized in a 20-μL reaction using a reverse transcriptase and oligo (dT) (TaKaRa Biotechnology., Ltd., Tokyo, Japan) or gene-specific stem-loop primers (Ribobio), whose sequences are all listed in Supplementary Table 3. The miR-181a, U6 and cel-miR-39-3p primers used in the study were obtained from Ribobio, and the others were synthesized by Sangon (Sangon Biotech Co., Ltd., Shanghai, China). RT-qPCR amplification was performed on a 7500 Real-Time PCR System (Applied Biosystems/Life Technologies, Grand Island, NY, USA). The relative expression levels of miR-181a in serum were normalized to those of cel-miR-39-3p, and the relative expression levels of miR-181a/mRNA in the liver and hepatocytes were normalized to those of U6/β-actin, whose cycles-to-threshold values were not affected by time, treatment, or interactions, findings that validated its usefulness as a control gene.

### Western blotting

Liver tissues or hepatocytes were lysed in lysis buffer (Beyotime) at 4 °C with PMSF. Total protein concentrations were estimated by BCA assay (Applygen Technologies Inc., Beijing, China), which was performed according to the manufacturer’s instructions. A total of 40 μg of protein was separated by 12% SDS-PAGE, transferred onto PVDF membranes (Millipore, Temecula, CA, USA), and immunoblotted with the appropriate primary antibodies. Then, the membranes were washed in Tris buffered saline with tween 20 (TBST) for 20 min, incubated with the appropriate horseradish peroxidase (HRP)-conjugated secondary antibodies (Protein Technology, Chicago, IL, USA) for 45 min at room temperature, and then washed in TBST for 15 min. The signals were subsequently detected using an ECL Kit (Millipore). All bands were analyzed using Image-pro Plus (Media Cybernetics, Silver Spring, MD).

### Luciferase assays

To generate reporter constructs, the complete 3’-UTR of human SIRT1 containing either wild-type or mutated miR-181a binding sites was cloned into pMIR-REPORT LUCIFERASE VECTOR (Ambion, Austin, TX, USA) behind the stop codon of the Firefly-luciferase open reading frame using specific primers. The integrity of the construct was verified using sequencing. HepG2 cells were transfected in 500 ng of DNA using X-tremeGENE HP DNA Transfection Reagent (Roche) according to the manufacturer’s instructions. Cells were harvested 48 h after transfection. Renilla and Firefly luciferase activity levels were measured consecutively using the Dual Luciferase Reporter Assay System (Promega corporation, Madison, USA) according to the manufacturer’s instructions.

### PGC-1α acetylation assays

For the PGC-1α acetylation assays, 1 mg of protein lysate was immunoprecipitated with PGC-1α for 10 h at 4 °C and then collected via the addition of protein A/G plus agarose beads (Santa Cruz) before being washed three times with lysis buffer (Beyotime). The precipitated proteins were analyzed by immunoblotting using an acetyl-lysine antibody.

### Measurement of hepatocyte and liver TG and glycogen contents

The TG content of hepatocyte and liver under the indicated conditions were measured using an enzymatic kit (Applygen Technologies Inc.) following the manufacturer’s instructions. Glycogen contents were measured using anthrone reagent (Jiancheng Bioengineering Institute, Nanjing, China), following the manufacturer’s instructions. Total protein concentration was estimated by the BCA method (Applygen Technologies Inc.) and performed according to manufacturer’s instructions. Hepatocytes and liver TG and glycogen levels were normalized to total protein contents.

### Measurement of glucose content in the medium

The glucose content of the medium treated as described above was measured using a glucose assay kit (Applygen Technologies Inc.). The cells were collected and lysed, and the total protein concentration was determined by BCA assay (Applygen Technologies Inc.) to correct for the cell counts.

### Statistical analysis

Data were expressed as the mean ± SD of at least three independent experiments. Statistical analysis was conducted using SPSS 19.0 software (SPSS Inc., Chicago, IL, USA), and the statistical significance (*P* < 0.05 was considered statistically significant) of comparisons involving two groups and more than two groups was calculated using Student′s *t* test and one-way ANOVA, respectively.

## SUPPLEMENTARY MATERIALS FIGURES AND TABLES




