# Supplementary material for: Upregulation of miR-181a impairs hepatic glucose and lipid homeostasis
Source: Oncotarget. 2017 Aug 24;8(53):91362–78. doi: 10.18632/oncotarget.20523 (PMC5710930; doi:10.18632/oncotarget.20523)
Supplement: Supplementary file 1 [file oncotarget-08-91362-s001.pdf]

Upregulation of miR-181a impairs hepatic glucose and lipid homeostasis

SUPPLEMENTARY MATERIALS

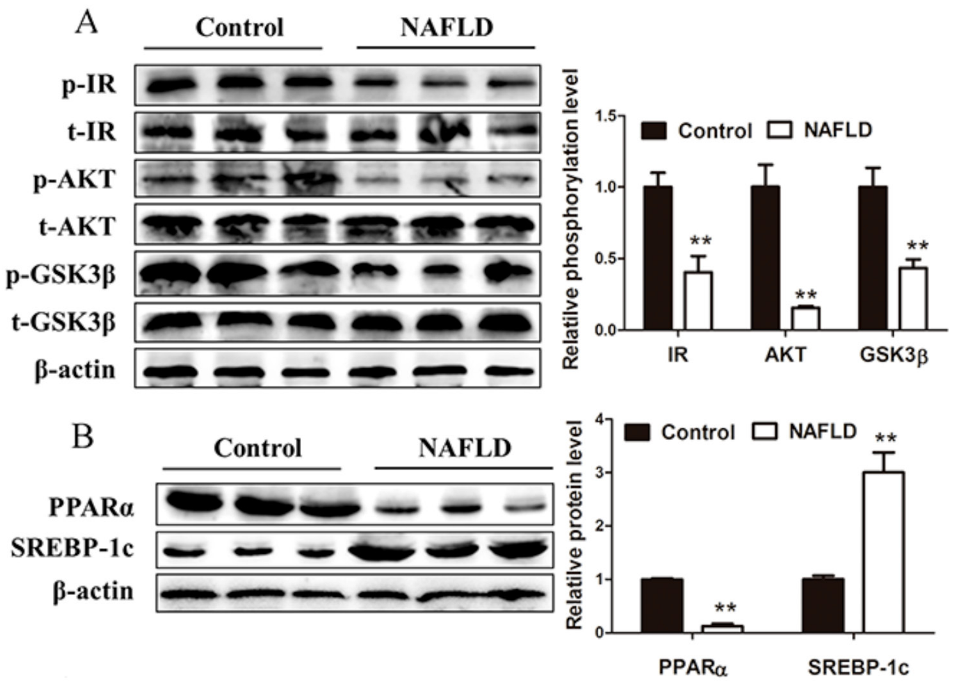

Supplementary Figure 1: The insulin-related and lipid-related IR/Akt/GSK3β and PARα/SREBP-1c signaling pathways. (A) Immunoblot analysis (left) and quantification (right) of insulin-stimulated phosphorylation of IR, Akt, GSK3β protein levels in the liver of dairy cows with NAFLD ($n = 20$) and controls ($n = 20$). **(B)** Immunoblot analysis (left) and quantification (right) of SREBP-1c and PPARα protein levels in the liver of dairy cows with NAFLD ($n = 20$) and controls ($n = 20$). $^{*}P < 0.05$, $^{**}P < 0.01$. All experiments were repeated at least three times and representative results are shown.

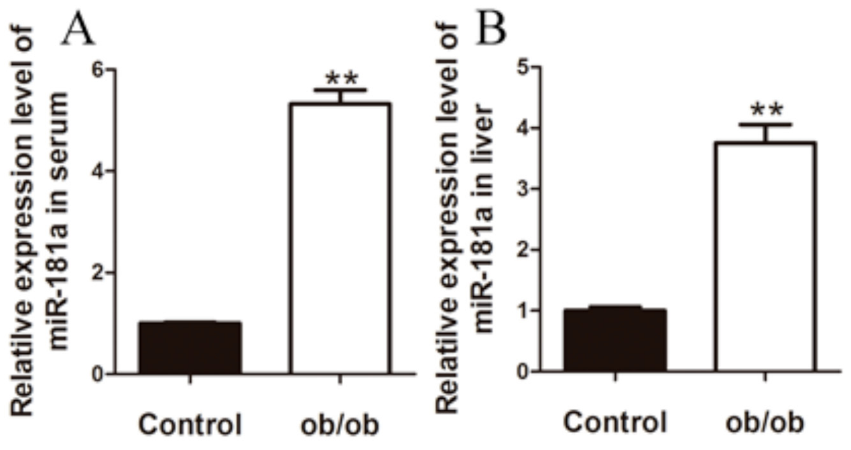

Supplementary Figure 2: Expression of miR-181a in ob/ob mice. (A) The expression level of miR-181a in the serum of ob/ob ($n = 7$) and control ($n = 7$) mice. **(B)** The expression level of miR-181a in the liver of ob/ob ($n = 7$) and control ($n = 7$) mice. $^{*}P < 0.05$, $^{**}P < 0.01$. All experiments were repeated at least three times and representative results are shown.

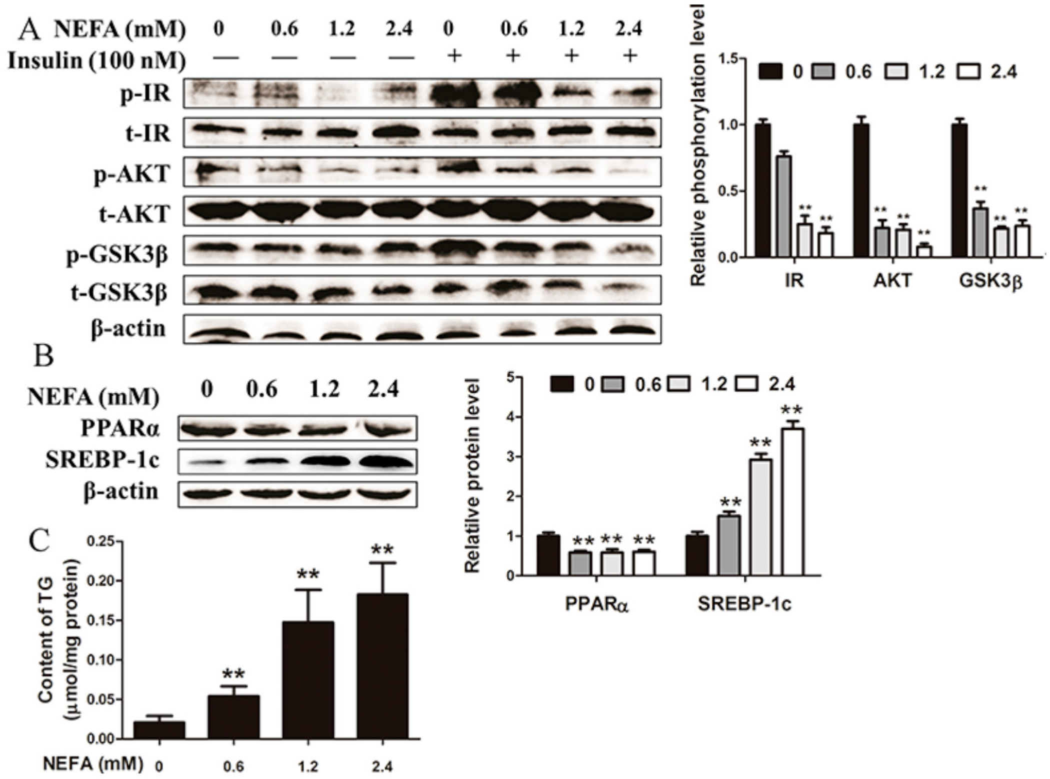

Supplementary Figure 3: High NEFA concentrations impair insulin signaling and lipid metabolism *in vitro*. (A) Immunoblot analysis (left) and quantification (right) of insulin-stimulated phosphorylation of IR, Akt, GSK3β protein levels in hepatocytes. **(B)** Immunoblot analysis (left) and quantification (right) of SREBP-1c and PPARα protein levels in hepatocytes. **(C)** TG content in hepatocytes. $^{*}P < 0.05$, $^{**}P < 0.01$. All experiments were repeated at least three times and representative results are shown.

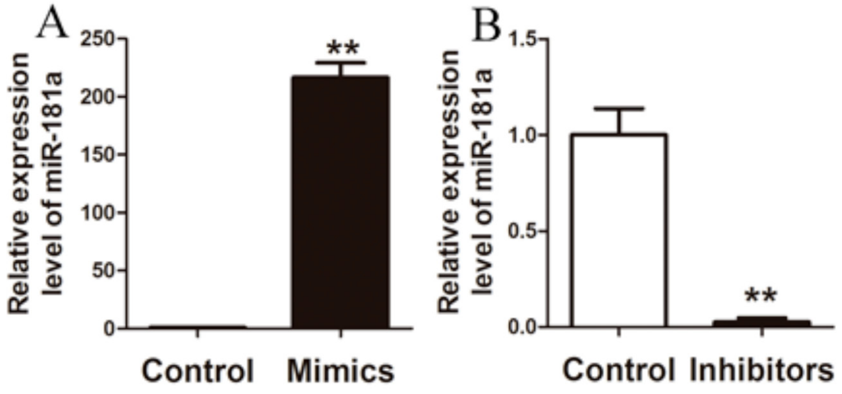

Supplementary Figure 4: Expression of miR-181a in dairy cow hepatocytes transfected with miR-181a mimics or inhibitors. $^{*}P < 0.05$, $^{**}P < 0.01$. All experiments were repeated at least three times and representative results are shown. **(A)** Hepatocytes were transfected with 10 nM miR-181a mimics or negative controls. **(B)** Hepatocytes were transfected with 50 nM miR-181a inhibitors or negative controls.

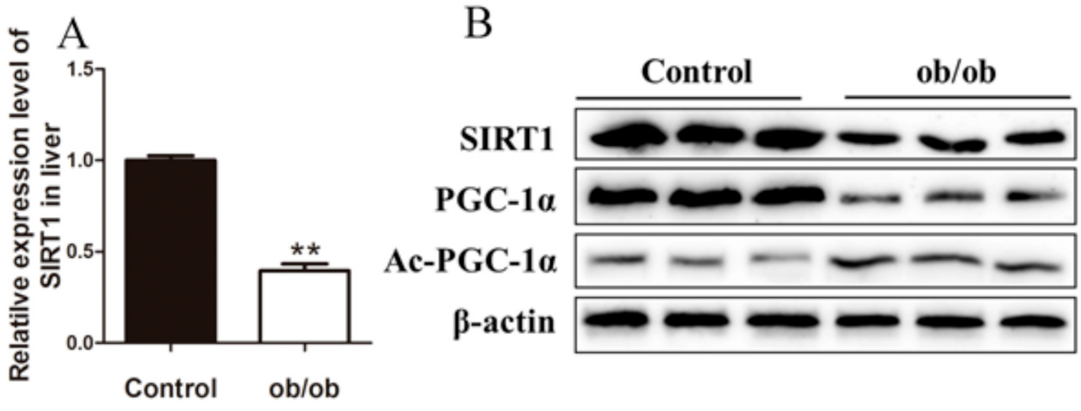

Supplementary Figure 5: The expression of SIRT1, PGC-1α and Ac-PGC-1α in the liver of ob/ob mice. **(A)** The mRNA expression level of SIRT1 in the liver of ob/ob ($n = 7$) and control ($n = 8$) mice. **(B)** Immunoblot analysis of SIRT1, PGC-1α and acetylated PGC-1α in the liver of ob/ob ($n = 7$) and control ($n = 7$) mice. $^{*}P < 0.05$, $^{**}P < 0.01$. All experiments were repeated at least three times and representative results are shown.

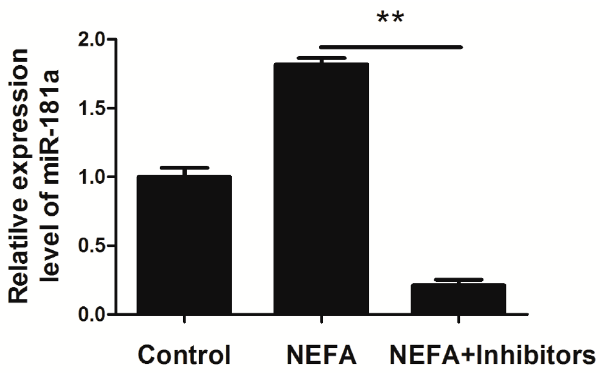

Supplementary Figure 6: The expression level of miR-181a in hepatocytes. Hepatocytes were divided into 3 groups as follows: a control group (transfected with 50 *nM* negative control), NEFA group (treated with 1.2 *mM* NEFA), and miR-181a + NEFA group (transfected with 50 *nM* miR-181a inhibitors and then treated with 1.2 *mM* NEFA). $^{*}P < 0.05$, $^{**}P < 0.01$. All experiments were repeated at least three times and representative results are shown.

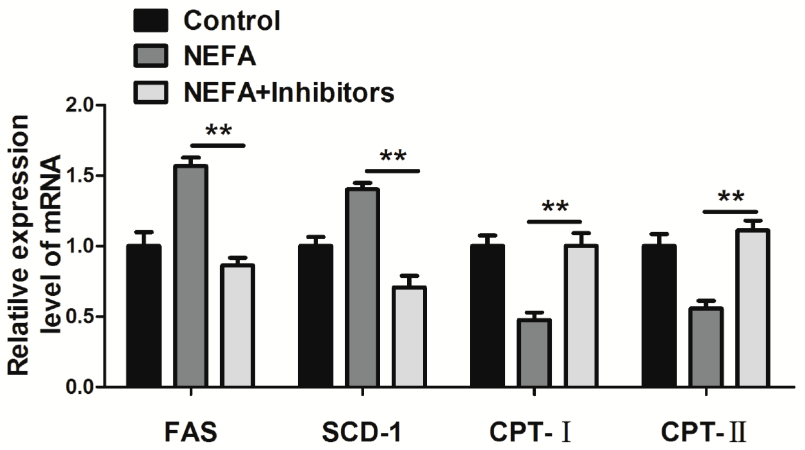

Supplementary Figure 7: The expression levels lipid metabolism genes in hepatocytes. Hepatocytes were divided into 3 groups as follows: a control group (transfected with 50 *nM* negative control), NEFA group (treated with 1.2 *mM* NEFA), and miR-181a + NEFA group (transfected with 50 *nM* miR-181a inhibitors and then treated with 1.2 *mM* NEFA). $^{*}P < 0.05$, $^{**}P < 0.01$. All experiments were repeated at least three times and representative results are shown.

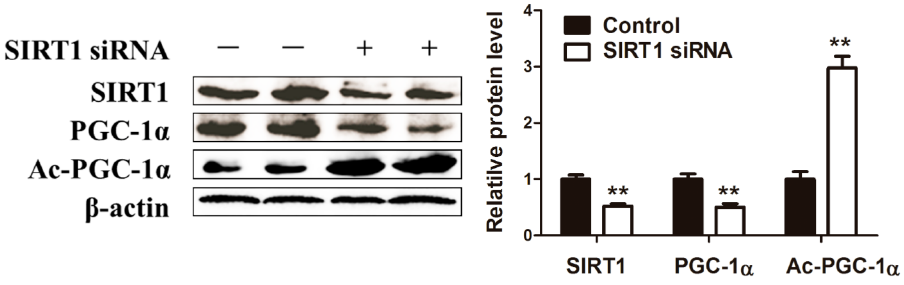

Supplementary Figure 8: Immunoblot analysis (left) and quantification (right) of SIRT1, PGC-1α and acetylated PGC-1α in hepatocytes. Hepatocytes were transfected with SIRT1 siRNA for 48 h and then harvested for WB. $^{*}P < 0.05$, $^{**}P < 0.01$. All experiments were repeated at least three times and representative results are shown.

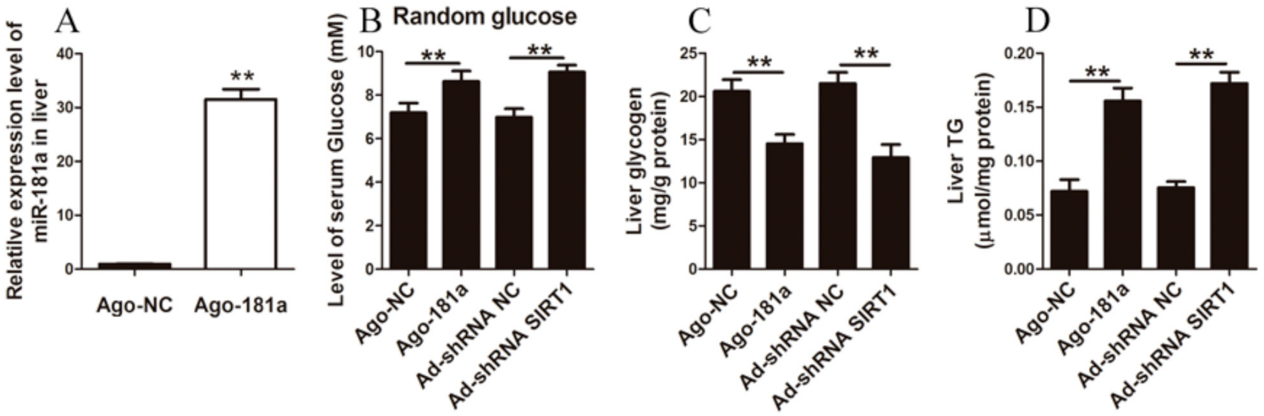

Supplementary Figure 9: Overexpression of miR-181a or knockdown of SIRT1 impairs glucose and lipid metabolism *in vivo*. **(A)** Relative miR-181a expression levels in the livers of mice injected with Ago-181a ($n = 8$) or Ago-NC ($n = 8$). **(B)** Random blood glucose levels of mice injected with Ago-181a ($n = 7$) or Ago-NC ($n = 7$) or Ad-shRNA NC ($n = 8$) or Ad-shRNA SIRT1 ($n = 8$). **(C)** The glycogen contents in the liver of mice injected with Ago-181a ($n = 7$) or Ago-NC ($n = 7$) or Ad-shRNA NC ($n = 8$) or Ad-shRNA SIRT1 ($n = 8$). **(D)** Liver TG contents in the mice injected with Ago-181a ($n = 7$) or Ago-NC ($n = 7$) or Ad-shRNA NC ($n = 8$) or Ad-shRNA SIRT1 ($n = 8$). $^{*}P < 0.05$, $^{**}P < 0.01$. All experiments were repeated at least three times and representative results are shown.

Supplementary Table 1: Basic description of the subjects (mean ± SD)

Variables	Control (n=15)	NAFLD (n=25)
Age (years)	47.2 ± 4.94	46.23 ± 8.45
Females/males	8/7	15/10
BMI (kg/m^2)	22.59 ± 1.47	29.88 ± 1.3**
NEFA (*mM*)	0.29 ± 0.08	0.96 ± 0.1**
Glucose (*mM*)	4.61 ± 0.46	5.87 ± 0.44**
Insulin (mU/L)	4.78 ± 0.52	11.00 ±1.89**
HbA_{1c} (%)	4.96 ± 0.61	7.83 ±0.8**
HbA_{1c} (mmol/mol)	30.6 ± 6.67	62.08 ± 8.76**
HOMA-IR	0.97 ± 0.14	2.84 ± 0.41**
TG (*mM*)	1.18 ± 0.25	2.34 ± 0.35**
ALT (IU/L)	22 ± 4.31	63.62 ± 8.21**
AST (IU/L)	18.50 ± 3.26	51.08 ± 5.08**
γ-GT (IU/L)	25.3 ± 5.02	91.84 ± 5.68**

*p<0.05 and **p<0.01 compared with control. BMI, Body mass index; HbA_{1c}, Hemoglobin A_{1c}; HOMA-IR, homeostasis model assessment of insulin resistance; TG, triglyceride; ALT, alanine aminotransferase; AST, aspartate transaminase; γ-GT, gamma-glutamyl transpeptidase.

Supplementary Table 2: Basic description of the dairy cows with NAFLD and controls (mean ± SD)

Variables	Control (n=20)	NAFLD (n=20)
Body weight (Kg)	562.26 ± 18.81	590.51 ± 15.82**
Body condition scores	2.59 ± 0.14	3.25 ± 0.25**
NEFA (*mM*)	0.27 ± 0.09	1.14 ± 0.2**
Glucose (*mM*)	3.82 ± 0.23	4.33 ± 0.27**
Insulin (mU/L)	15.29 ± 1.08	22.19 ± 1.67**
ALT (IU/L)	20.81 ± 2.71	36.80 ± 4.46**
AST (IU/L)	42.85 ± 3.4	103.87 ± 10.98**
γ-GT (IU/L)	19.59 ± 2.97	28.26 ± 1.79**

*p<0.05 and **p<0.01 compared with healthy cows. NEFA, non-esterified fatty acids; ALT, Alanine aminotransferase; AST, Aspartate transaminase; γ-GT, gamma-glutamyl transpeptidase.

Supplementary Table 3: Primers for Real-time PCR

See Suppleementary File 1
