# Supplementary material for: Upregulation of miR-181a impairs hepatic glucose and lipid homeostasis
Source: Oncotarget. 2017 Aug 24;8(53):91362–78. doi: 10.18632/oncotarget.20523 (PMC5710930; doi:10.18632/oncotarget.20523)
Supplement: Supplementary file 2 [file oncotarget-08-91362-s002.docx]

**Supplementary Table 3: Primers for Real-time PCR**

| Name | Sequence (5’-3’) |
| --- | --- |
| U6 RT primer | CTCAACTGGTGTCGTGGAGTCGGC  AATTCAGTTGAGATATGGAA |
| U6 forward | ACACTCCAGCTGGGATTCGTGAAGCG |
| U6 reverse | CTCAACTGGTGTCGTGGAGTCGG |
| Cel-miR-39-3p forward | ATGGTTCGTGGGTCACCGGGTGTAAATC |
| Cel-miR-39-3p reverse | GCAGGGTCCGAGGTATTC |
| miR-181a RT primer | GTCGTATCCAGTGCAGGGTCCGAGGT  ATTCGCACTGGATACGACAACTCAC |
| miR-181a forward | GCGGCGAACATTCAACGATG |
| miR-181a reverse | GTGCAGGGTCCGAGG |
| Bta-β-actin forward | GCCCTGAGGCTCTCTTCCA |
| Bta-β-actin reverse | GCGGATGTCGACGTCACA |
| Hsa-β-actin forward | GCTAACAGTCCGCCTAGAAGCA |
| Hsa-β-actin reverse | GTCATCACCATCGGCAATGAG |
| Bta-SIRT1 forward | ATACACTGGAGCAGGTT |
| Bta-SIRT1 reverse | TTCATCAGCTGGGCATCTAG |
| Bta-G6Pase forward | AGCAAGTGGTTCCCGTTTC |
| Bta-G6Pase reverse | ACCCAGGCGAGGCAGTA |
| Hsa-G6Pase forward | CCCAGGTTCACCAGTTCCC |
| Hsa-G6Pase reverse | GCCGTCATTATGGGCCAGA |
| Bta-PEPCK forward | AAGTACCTTGAGGAGCAAGTGAA |
| Bta-PEPCK reverse | GGTGCGTTGTATGGATTGGA |
| Has-PEPCK forward | AAAACGGCCTGAACCTCTCG |
| Has-PEPCK reverse | ACACAGCTCAGCGTTATTCTC |
| Bta-FAS forward | CAGCTTTGTGTTGGCAGAGAAG |
| Bta-FAS reverse | AGCGAGCTGTCCAGGTTGAC |
| Bta-SCD-1 forward | AGCGAGCTGTCCAGGTTGAC |
| Bta-SCD-1 reverse | CAGCCACTCTTGTAGCTTTCCTC |
| Bta-CPT-Ⅰ forward | ACGCCGTGAAGTATAACCCT |
| Bta-CPT-Ⅰ reverse | CCAAAAATCGCTTGTCCCTT |
| Bta-CPT-Ⅱ forward | TGAACATCCTCTCCATCTGG |
| Bta-CPT-Ⅱ reverse | GGTCAACAGCAACTACTACG |
